# Interpersonal Emotion Regulation in Middle Childhood: From Cognitive Capacity to Context-Sensitive Regulation

**DOI:** 10.3390/bs16101862

**Published:** 2026-10-09

**Authors:** Jiaqi Zhang, Chenxi Deng, Wen Liu

**Affiliations:** 1 College of Psychology, Liaoning Normal University, 850 Huanghe Road, Sha Hekou District, Dalian 116029, China; zjq_0222@126.com (J.Z.); dengchenxi@lnu.edu.cn (C.D.); 2 Psychological Counseling Center, Liaoning University, Shenyang 110036, China

**Keywords:** interpersonal emotion regulation, middle childhood, executive function, empathy, theory of mind, context sensitivity, regulatory goals, regulatory demands

## Abstract

Interpersonal emotion regulation (IER) requires individuals to understand another person’s emotional state and adapt regulatory decisions to interpersonal goals and situational demands. Across four linked studies, the present research examined age-related patterns, cognitive and social-cognitive correlates, contextual modulation, and individual differences in context sensitivity in children aged 8–10 years. IER was assessed using vignette-based strategy ratings, executive-function and social-cognitive measures, and experimental manipulations of regulatory goals and demands. Across the observed age range, increasing age was associated with greater cognitive and affective engagement and lower avoidance and co-rumination, whereas distraction and suppression showed no significant age associations. Executive function, empathy, and theory of mind were systematically associated with greater adaptive and lower maladaptive IER. Children also altered their regulatory decisions as social goals and regulatory demands changed. Person-by-context analyses showed selective effects: general cognitive-control capacity moderated responsiveness to regulatory goals, whereas social cognition moderated demand-related variation in cognitive engagement. Together, the findings suggest that IER in middle childhood involves not only developing regulatory capacities but also coordinating those capacities with the goals and demands of interpersonal situations.

## 1. Introduction

Interpersonal emotion regulation (IER) refers to processes through which individuals intentionally influence another person’s emotional experience or expression during social interaction (Reeck et al., 2016; Zaki & Williams, 2013). Unlike intrapersonal emotion regulation, which concerns the management of one’s own emotions, other-focused IER requires the regulator to perceive another person’s emotional state, determine whether and how that state should be altered, and select a response appropriate to the ongoing interaction. Although IER can overlap behaviorally with supportive or prosocial responding, similar behaviors constitute IER when they are directed toward altering the target’s emotional state, whereas they may otherwise serve broader supportive or prosocial goals (López-Pérez et al., 2016; Nozaki & Gross, 2025). These processes are relevant to social support, affiliation, relationship functioning, and goal pursuit (Blair et al., 2016; Williams et al., 2018). Recent theoretical developments increasingly conceptualize IER not as a single regulatory act, but as a dynamic interpersonal process involving the regulator, the target, and the goals and constraints of the situation (Arican-Dinc & Gable, 2025; Niven & López-Pérez, 2025; Nozaki & Gross, 2025). Developmental evidence further suggests that children become increasingly capable of using cognitively and emotionally elaborated strategies to influence others’ emotions across childhood (López-Pérez et al., 2016; López-Pérez & Pacella, 2021). Middle childhood may therefore represent an important period for examining not only age-related variation in IER, but also the developing capacities and contextual processes that shape children’s regulatory choices (Antony et al., 2025). In the present research, other-focused IER was examined specifically in child-to-peer contexts, consistent with developmental work examining children’s response strategies within friendships (Rose & Asher, 2004). Across the present paradigms, the regulatory target was a best friend, partner, or opponent described as another child rather than an adult.

Process-oriented accounts provide a useful framework for understanding these developmental changes. The social regulatory cycle conceptualizes other-focused emotion regulation as a sequence of operations in which the regulator represents the target’s current emotional state, evaluates the need for regulation, establishes a desired emotional outcome, selects a regulatory strategy, and implements that strategy (Reeck et al., 2016). Contemporary process accounts similarly emphasize identification, selection, implementation, and monitoring as central components of other-focused emotion regulation (Nozaki & Gross, 2025). These models imply that IER depends on complementary domain-general cognitive and social-cognitive capacities. Executive functions—including inhibitory control, working memory, and cognitive flexibility—may support the maintenance of regulatory goals, inhibition of ineffective responses, coordination of relevant information, and adjustment of behavior as task demands change (Diamond, 2013; Hudson & Jacques, 2014; Simonds et al., 2007). Empathy and theory of mind (ToM), in contrast, may support the representation of another person’s emotional experience, beliefs, intentions, and perspective (Zaki, 2020). More broadly, individual-differences accounts of IER have identified cognitive abilities as potential contributors to successful regulation at multiple stages of the interpersonal regulatory process (Niven et al., 2024), while developmental research has linked ToM and cognitive empathy to other-oriented social behavior in children (Zhang et al., 2023). Together, these perspectives suggest that general cognitive-control capacity and social cognition may provide complementary capacities for regulating others’ emotions. Having the capacity to regulate, however, does not determine what emotional state the regulator should attempt to produce.

This distinction between regulatory capacity and regulatory goal is central to motivational approaches to emotion regulation. Emotion regulation is goal directed, and desired emotional states can serve broader motives that extend beyond the immediate pursuit of pleasure or avoidance of distress (Tamir, 2016). Accordingly, a distinction can be made between hedonic goals, which prioritize immediate affective improvement, and instrumental goals, for which an emotional state is valued because of its anticipated usefulness for achieving another objective. This distinction also applies to the regulation of other people’s emotions. Regulators may sometimes seek to improve another person’s immediate emotional experience, but at other times may prefer an emotional state because it facilitates—or interferes with—the target’s performance in ways that are relevant to the regulator’s own goal (Netzer et al., 2015; Niven, 2016). Thus, understanding another person’s emotion and wanting that person to feel better are theoretically distinct processes. A child may understand another person’s emotional state accurately yet prefer a different emotional outcome depending on whether that person is a cooperative partner or a competitor and on whether a particular emotion is useful for the task at hand. This raises an important developmental question: can children coordinate their understanding of another person’s emotions with the instrumental structure of an ongoing social interaction? Experimental evidence further shows that anger can improve performance in confrontational tasks (Tamir et al., 2008), while interpersonal work demonstrates that people adjust the emotions they seek to induce in partners versus rivals according to whether those emotions are useful for the other person’s task performance (Netzer et al., 2015).

Regulatory goals are only one source of contextual variation. Even when the desired emotional outcome is clear, the functional value of a regulatory strategy may depend on the demands of the situation. Regulatory-flexibility accounts challenge the assumption that specific emotion-regulation strategies are uniformly adaptive or maladaptive, emphasizing instead the fit between regulatory responses and contextual requirements (Bonanno & Burton, 2013). A related person–situation–strategy perspective proposes that regulatory outcomes depend jointly on characteristics of the individual, the situation, and the strategy being deployed (Doré et al., 2016). This contextual approach is increasingly relevant to IER. For example, when another person’s negative emotion requires rapid down-regulation, regulators may shift away from emotionally engaging responses and toward strategies such as distraction or suppression that facilitate more immediate disengagement from the emotional experience (Pauw et al., 2019). More recent work further demonstrates that the perceived usefulness and outcomes of IER strategies can differ according to whether regulation serves affective improvement or instrumental goal pursuit (Pauw et al., 2026). Such findings support a more context-sensitive view in which regulatory responses are evaluated relative to what the situation requires rather than solely according to their average or habitual consequences. Developmental research has shown that children can exhibit flexibility in their own emotion-regulation strategy use (Parsafar et al., 2019), but considerably less is known about whether children adapt other-focused regulatory choices to changing interpersonal goals and situational demands. In the present research, we use context sensitivity to refer specifically to systematic variation in children’s IER choices as a function of such contextual features. Context sensitivity is therefore treated as one component of broader regulatory flexibility rather than as evidence of the full flexibility construct, which also encompasses strategy repertoire, dynamic switching, feedback responsiveness, and ongoing monitoring.

Within the vignette-based measurement framework used in Studies 1 and 2, cognitive engagement, affective engagement, and distraction were grouped as adaptive IER, whereas suppression, avoidance, and co-rumination were grouped as maladaptive IER, following the task’s prespecified classification framework (López-Pérez & Pacella, 2021), which was informed more broadly by prior classifications of controlled interpersonal affect-regulation strategies (Niven et al., 2009). These categories provide a task-based descriptive organization of regulatory responses and are not assumed to represent the context-invariant functional properties of the strategies.

Bringing these perspectives together suggests that the development of IER cannot be fully understood by examining either individual capacity or social context in isolation. Process accounts identify the operations required to regulate another person; cognitive and social-cognitive perspectives identify capacities that may support these operations; motivational approaches specify the emotional outcomes that regulators seek; and regulatory-flexibility perspectives emphasize that appropriate regulatory responses depend on situational demands. Their integration leads to a further question concerning person-by-context variation. Children may differ not only in their general tendency to use particular interpersonal regulatory strategies but also in the extent to which their choices change as social goals and regulatory demands change. Stronger executive function may facilitate maintaining a context-specific regulatory objective and adjusting responses when task demands differ, whereas empathy and ToM may contribute to representing how the target’s emotional state and regulatory needs vary across situations. From this perspective, cognitive capacity should not necessarily be expected to relate to IER in a context-invariant manner. Rather, developing capacities may also be associated with the degree to which children adapt their regulatory decisions to the interpersonal circumstances in which regulation occurs. This possibility links individual-differences approaches to the broader question of context-sensitive regulation and provides a developmental extension of person–situation perspectives on emotion regulation.

The present research examined IER in children aged 8–10 years by integrating developmental, cognitive, and contextual perspectives. First, we examined age-related patterns in children’s use of interpersonal regulatory strategies during middle childhood. Second, we examined whether executive function, including inhibitory control, working memory, and cognitive flexibility, and social cognition, including empathy and ToM, were associated with children’s use of adaptive and maladaptive IER strategies. Third, we experimentally examined contextual modulation by varying regulatory goals and regulatory demands. Regulatory-goal conditions tested whether children’s preferred emotional outcomes changed according to cooperative versus competitive relationships and according to the instrumental value of happiness and anger for task performance. Regulatory-demand conditions tested whether the urgency of another person’s need for emotional down-regulation altered children’s preferences among engagement, distraction, and suppression-based strategies. Finally, we examined person-by-context effects at two broader levels of individual capacity: general cognitive-control capacity, indexed by a composite integrating inhibitory control, working memory, and cognitive flexibility, and social cognition, indexed by a composite integrating empathy and ToM. Taken together, these theoretical and empirical considerations led to four sets of hypotheses. First, increasing age across the observed range was expected to be associated with greater cognitive and affective engagement and lower suppression, avoidance, and co-rumination, whereas distraction was not expected to show a significant age association (H1). Second, stronger inhibitory control, working memory, cognitive flexibility, empathy, and ToM were expected to be associated with a greater use of adaptive and lower use of maladaptive IER strategies (H2). Third, children’s regulatory choices were expected to vary with social goals and situational demands. Under cooperation, children were expected to prefer happiness in an approach-oriented task and anger in an aggression-oriented task, whereas the reverse pattern was expected under competition; when hedonic and instrumental considerations conflicted, instrumental utility was expected to predominate (H3a). Under high regulatory demand, children were expected to use more distraction and suppression and less cognitive and affective engagement, with the reverse pattern expected under low demand (H3b). Finally, general cognitive-control capacity and social cognition were expected to interact with regulatory goals and regulatory demands in predicting children’s IER. Given the limited evidence concerning the form of these interactions during middle childhood, these interaction hypotheses were nondirectional (H4).

## 2. Study 1: Age-Related Patterns of Interpersonal Emotion Regulation

Study 1 examined age-related patterns in children’s interpersonal emotion regulation (IER) during middle childhood. A vignette-based task was used to assess children’s reported regulatory responses toward a close peer experiencing a socially distressing event.

### 2.1. Materials and Methods

#### 2.1.1. Participants

The original a priori sample-size calculation was conducted using G*Power 3.1 (Faul et al., 2007) for a one-way ANOVA with three age groups. Assuming an effect size of f = 0.25, α = 0.05, and power (1 − β) = 0.80, the calculation indicated a minimum total sample size of 159. This calculation served as the original recruitment benchmark. The final sample consisted of 220 children aged 8–10 years recruited from an urban elementary school. Children were grouped into three age bands: 8 years to 8 years 6 months, 9 years to 9 years 6 months, and 10 years to 10 years 6 months. Efforts were made to achieve a comparable distribution of boys and girls across age groups. All participating children were reported to be in good physical health and to have normal vision.

The 8-year-old group included 72 children (36 boys and 36 girls; *M* age = 101.42 months, *SD* = 3.51), the 9-year-old group included 76 children (38 boys and 38 girls; *M* age = 113.41 months, *SD* = 3.71), and the 10-year-old group included 72 children (35 boys and 37 girls; *M* age = 125.10 months, *SD* = 3.73). Overall, the sample comprised 109 boys and 111 girls.

The prespecified age bands reflected the original cross-sectional sampling framework rather than theoretically discrete developmental stages; sampling selected developmental points is an established approach in developmental research (Adolph & Robinson, 2011).

All studies reported in this article were approved by the Ethics Committee of Liaoning Normal University. Written informed consent was obtained from the children’s guardians, and school/teacher permission was obtained for conducting the research in the school setting. After the study was introduced to the children, participation was voluntary; children who indicated willingness to take part were enrolled only after written guardian consent had been obtained. A separate written child-assent form was not collected.

#### 2.1.2. Procedure

The IER assessment was adapted from previously used vignette-based paradigms (Pauw et al., 2019; Rose & Asher, 2004). Children were presented with a hypothetical social stress scenario involving their best friend. In the scenario, the friend was required to give a presentation in front of the class, forgot what to say, performed poorly, and was subsequently laughed at and teased by several classmates. The scenario ended at the end of the school day, when the participant would normally spend time with the friend. The complete vignette is provided in the Supplementary Materials.

The vignette was adapted from established paradigms rather than developed de novo, retaining the central interpersonal-regulation structure while situating the event in a concrete, age-relevant peer and school context. Response options were similarly presented as concrete interpersonal behaviors rather than abstract strategy labels. These design features are consistent with recommendations emphasizing theoretically grounded construction, realism, and transparent reporting in vignette research (Aguinis & Bradley, 2014; Hillen et al., 2025).

After the vignette, children first identified how they thought their friend was feeling by choosing among five emotional states: happy, sad, afraid, angry, and surprised. They were then asked to consider how they would respond to their friend following the event. The task presented 18 concrete interpersonal response items rather than abstract strategy labels. Children rated each response on a 7-point Likert-type scale ranging from 1 (not at all) to 7 (very much). Representative responses included giving the friend advice, offering comfort, and suggesting an enjoyable activity. Because the task was administered at the level of concrete responses, children were not required to learn or distinguish the researcher-defined strategy labels.

#### 2.1.3. Interpersonal Emotion Regulation Strategies

For scoring, the response items were assigned a priori to strategy categories according to the task framework, drawing on prior classifications of children’s IER and controlled interpersonal affect regulation (López-Pérez & Pacella, 2021; Niven et al., 2009). Cognitive engagement, affective engagement, and distraction each comprised four items. Cognitive engagement involved attempts to modify how the target interpreted the stressful situation, such as providing advice or emphasizing supportive aspects of the situation. Affective engagement involved directly addressing the target’s emotional experience through comfort, listening, or conversation. Distraction involved redirecting the target’s attention away from the distressing event toward a different or more pleasant activity. For example, a distraction item asked children to indicate the extent to which they would ask their friend to play a game or do something enjoyable, whereas an avoidance item asked the extent to which they would act as though the teasing incident had never occurred.

Suppression, avoidance, and co-rumination each comprises two items. Suppression involved attempts to inhibit or discourage the target’s emotional response or expression. Avoidance involved disregarding the negative emotional event or encouraging the target to disengage from the negative emotional experience. Co-rumination involved extended engagement with the target’s worries and negative emotional content in ways that could maintain or intensify distress.

Ratings were summed within each strategy category. Cognitive engagement, affective engagement, and distraction therefore had possible scores of 4–28, whereas suppression, avoidance, and co-rumination had possible scores of 2–14. The overall adaptive IER index summed cognitive engagement, affective engagement, and distraction (12 items; possible range 12–84), and the overall maladaptive IER index summed suppression, avoidance, and co-rumination (6 items; possible range 6–42). The strategy labels were researcher-defined analytic categories for organizing concrete vignette responses; children were not required to classify the responses using these abstract terms. In Study 1, the six strategy-specific scores were treated as the primary outcomes, whereas the adaptive and maladaptive indices were retained as secondary task-based summary composites.

#### 2.1.4. Statistical Analysis

Data were managed using Microsoft Excel, and the age-related analyses were conducted using Python 3.13.5 with statsmodels 0.14.6. Descriptive statistics were calculated for children’s emotion-identification responses and IER strategy scores. Age-related associations in the six IER strategies were examined using ordinary least-squares regression with children’s exact age in months entered as a continuous predictor. HC3 heteroscedasticity-robust standard errors were used for statistical inference. The six strategy-specific age effects were treated as a single family of tests and adjusted using the Benjamini–Hochberg false discovery rate (FDR) procedure. The overall adaptive and maladaptive IER indices were analyzed as secondary composite outcomes and were not included in this FDR family. Effect estimates are reported as unstandardized coefficients rescaled to represent a 12-month increase in age, together with 95% confidence intervals, standardized β coefficients, and R^2^ values. Sensitivity analyses added sex as a covariate and tested a quadratic age term. One affective-engagement score fell outside the specified response range and was treated as missing for that outcome; the corresponding overall adaptive IER score was therefore also treated as missing. Statistical significance was evaluated at α = 0.05.

### 2.2. Results

#### 2.2.1. Emotion Identification

Across the full sample, 87.3% of children identified sadness as the target’s emotional state following the vignette. Descriptively, the proportion identifying sadness was 81.9% among 8-year-olds, 85.5% among 9-year-olds, and 94.4% among 10-year-olds, indicating that most children in each age band identified the intended negative emotional state.

#### 2.2.2. Age-Related Patterns in Adaptive IER

Continuous-age models for adaptive IER are presented in Table 1. Cognitive engagement was positively associated with age: for each 12-month increase in age, cognitive-engagement scores were estimated to increase by 2.05 points, where 95% CI [1.42, 2.69], β = 0.379, R^2^ = 0.144, *p* < 0.001, FDR-adjusted q < 0.001. Affective engagement showed a similar positive association with age, where B = 1.79 points per 12 months, 95% CI [1.23, 2.35], β = 0.396, R^2^ = 0.157, *p* < 0.001, q < 0.001.

Distraction was not significantly associated with age, with B = 0.27 points per 12 months, 95% CI [−0.55, 1.09], β = 0.044, R^2^ = 0.002, *p* = 0.519, q = 0.519. The overall adaptive IER index, analyzed as a secondary composite outcome, was positively associated with age, where B = 4.09 points per 12 months, 95% CI [2.55, 5.63], β = 0.324, R^2^ = 0.105, *p* < 0.001.

#### 2.2.3. Age-Related Patterns in Maladaptive IER

Continuous-age models for maladaptive IER are presented in Table 2. Suppression was not significantly associated with age, where B = 0.27 points per 12 months, 95% CI [−0.14, 0.68], β = 0.083, R^2^ = 0.007, *p* = 0.199, q = 0.239. In contrast, avoidance was negatively associated with age, where B = −0.84 points per 12 months, 95% CI [−1.33, −0.34], β = −0.224, R^2^ = 0.050, *p* < 0.001, q = 0.002.

Co-rumination was also negatively associated with age, where B = −0.81 points per 12 months, 95% CI [−1.37, −0.24], β = −0.188, R^2^ = 0.035, *p* = 0.005, q = 0.007. The overall maladaptive IER index, analyzed as a secondary composite outcome, was negatively associated with age, where B = −1.38 points per 12 months, 95% CI [−2.49, −0.26], β = −0.160, R^2^ = 0.026, *p* = 0.016.

#### 2.2.4. Sensitivity Analyses and Hypothesis Evaluation

Adding sex as a covariate did not alter the direction, magnitude, or statistical inference of the six strategy-specific age effects. Quadratic age terms were nonsignificant for all six strategies after FDR correction (all q = 0.932), providing no evidence that a nonlinear age specification improved the models. Retaining the out-of-range affective-engagement value in a sensitivity analysis likewise did not change the inference for affective engagement or the overall adaptive IER index.

Overall, H1 was partially supported. Increasing age was associated with greater cognitive and affective engagement and with lower avoidance and co-rumination. Distraction and suppression showed no significant age associations; therefore, the predicted age-related decrease in suppression was not supported.

## 3. Study 2: Cognitive and Social-Cognitive Correlates of Interpersonal Emotion Regulation

Study 2 examined whether individual differences in cognitive and social-cognitive capacities were associated with children’s IER. Study 2a focused on three components of executive function—inhibitory control, working memory, and cognitive flexibility—whereas Study 2b examined empathy and theory of mind (ToM). Together, these analyses tested whether capacities supporting cognitive control and the representation of others’ emotional and mental states were associated with children’s reported tendencies to use adaptive and maladaptive IER strategies.

### 3.1. Study 2a: Executive-Function Correlates

#### 3.1.1. Materials and Methods

**Participants.** The original a priori sample-size calculation was conducted using G*Power 3.1 (Faul et al., 2009) for a two-tailed test of a correlation coefficient. Assuming r = 0.30, α = 0.05, and power (1 − β) = 0.80, the calculation indicated a minimum sample size of 82. This calculation served as the original recruitment benchmark for detecting associations between executive-function measures and IER. The final sample consisted of 125 children aged 8–10 years recruited from an urban elementary school. The 8-year-old group included 47 children (24 boys and 23 girls; *M* age = 100.85 months, *SD* = 3.67), the 9-year-old group included 42 children (22 boys and 20 girls; *M* age = 113.19 months, *SD* = 3.49), and the 10-year-old group included 36 children (19 boys and 17 girls; *M* age = 125.36 months, *SD* = 3.17). Overall, the sample comprised 65 boys and 60 girls. All participating children were reported to be in good physical health and to have normal vision.

**Interpersonal emotion regulation.** The IER assessment was identical to that used in Study 1. Children evaluated how they would respond to a distressed best friend in the same hypothetical social-stress vignette and rated the concrete regulatory-response items using the same 7-point format. The task-based adaptive and maladaptive summary indices described in Study 1 served as the outcome variables in the present analyses.

**Inhibitory control.** Inhibitory control was assessed using a computerized word–color Stroop task (van Veen & Carter, 2005), administered using E-Prime 2.1. Children were instructed to identify the font color of Chinese color words while ignoring the semantic meaning of the word. The stimuli involved the words corresponding to red, green, and blue, presented in congruent and incongruent font colors. Following practice, the task comprised 100 test trials presented in random order. Each trial included a 500 ms fixation, a 500 ms stimulus presentation, and a 1000 ms interstimulus interval. Accuracy and response time were recorded. Following a composite-scoring approach used in child cognitive-task research (Milward et al., 2017), accuracy and mean response time were standardized across participants, the direction of the standardized response-time score was reversed, and the two standardized scores were summed. Higher composite scores therefore reflected the joint contribution of greater accuracy and faster responding.

**Working memory.** Working memory was assessed using an adapted computerized task based on Steenari et al. (2003). On each trial, children viewed a yellow dot appearing at one of four locations and judged whether its location matched that shown on the preceding trial. Stimuli were presented using E-Prime 2.1. Each trial consisted of a 500 ms fixation, a 500 ms stimulus presentation, and a 1000 ms interstimulus interval. Children proceeded to the test trials after reaching at least 80% accuracy during practice. Accuracy and response time were recorded. The same composite-scoring procedure was used, combining standardized accuracy with sign-reversed standardized mean response time so that higher values reflected more accurate and faster task performance.

**Cognitive flexibility.** Cognitive flexibility was assessed using a computerized Flanker paradigm (Diamond et al., 2007) implemented in E-Prime 2.1. The task included standard, reversed, and mixed conditions. In the standard condition, children responded according to the direction of the central blue fish; in the reversed condition, they responded according to the direction of the flanking pink fish. In the mixed condition, blue and pink stimuli were presented in random order, requiring children to switch between the two response rules. The standard and reversed conditions each contained 4 practice trials and 17 test trials, and the mixed condition contained 4 practice trials and 45 test trials. Cognitive-flexibility performance was indexed by accuracy and mean correct-response RT from the mixed condition, in which blue and pink trials were intermixed, requiring children to switch between the two response rules according to stimulus color. Mixed-block accuracy and RT were combined using the standardized composite procedure described above. The resulting score was interpreted as a broad index of flexible rule-switching performance rather than as a pure trial-level switch-cost measure.

Executive-function scoring. For all three executive-function tasks, mean RT was calculated from correct-response trials only. RT values exceeding 3 SD above the relevant mean were excluded before participant-level mean RTs were calculated. Accuracy and mean correct-response RT were then standardized across participants; the standardized RT score was sign-reversed and summed with standardized accuracy, such that higher composite scores reflected the joint contribution of greater accuracy and faster correct responding. This scoring approach follows the speed–accuracy composite procedure used by Milward et al. (2017).

**Procedure.** Children were tested individually. Before formal testing, the experimenter interacted with children in their classroom to reduce unfamiliarity. The IER assessment preceded the relevant executive-function assessment in each testing sequence.

**Statistical analysis.** Data were managed using Microsoft Excel. Component-level associations were analyzed using Python 3.13.5 with statsmodels 0.14.6. Inhibitory control, working memory, and cognitive flexibility were examined in separate ordinary least-squares regression models predicting adaptive and maladaptive IER, with exact age in months included as a continuous covariate. HC3 heteroscedasticity-robust standard errors were used for statistical inference. The six executive-function component–IER coefficients were treated as a single family of tests and adjusted using the Benjamini–Hochberg false discovery rate (FDR) procedure. Unstandardized coefficients, 95% confidence intervals (CIs), standardized β coefficients, R^2^, and incremental ΔR^2^ values are reported. Sex was added in sensitivity analyses. The three executive-function components were also modeled as indicators of a latent executive-function factor in Mplus 7.4 using maximum-likelihood estimation. Separate latent-factor models predicted adaptive and maladaptive IER. These SEMs were estimated without additional covariates.

#### 3.1.2. Results

Associations between executive-function components and IER are summarized in Table 3. After adjustment for exact age, inhibitory control was positively associated with adaptive IER, with B = 3.58, 95% CI [0.78, 6.38], β = 0.260, *p* = 0.012, FDR-adjusted q = 0.015, and negatively associated with maladaptive IER, where B = −2.38, 95% CI [−4.10, −0.65], β = −0.275, *p* = 0.007, q = 0.010.

Working memory was positively associated with adaptive IER, where B = 5.03, 95% CI [2.64, 7.42], β = 0.390, *p* < 0.001, q < 0.001, and negatively associated with maladaptive IER, where B = −1.87, 95% CI [−3.46, −0.28], β = −0.231, *p* = 0.021, q = 0.021.

Cognitive flexibility showed the strongest standardized associations: it was positively associated with adaptive IER, with B = 3.35, 95% CI [2.05, 4.64], β = 0.433, *p* < 0.001, q < 0.001, and negatively associated with maladaptive IER, where B = −2.33, 95% CI [−3.49, −1.17], β = −0.416, *p* < 0.001, q < 0.001.

Task performance was also examined descriptively. Mean accuracy (SD) and mean response time in milliseconds (SD) were 0.691 (0.142) and 593.35 (107.58) for inhibitory control, 0.688 (0.127) and 771.41 (125.28) for working memory, and 0.843 (0.096) and 731.32 (77.06) for cognitive flexibility. At the participant level, accuracy was positively associated with mean response time for inhibitory control, r = 0.455, q < 0.001, and working memory, r = 0.378, q < 0.001, whereas the association was not significant for cognitive flexibility, r = −0.174, q = 0.053. These patterns are compatible with a speed–accuracy trade-off in the first two tasks and support interpreting the composite scores as joint indices of accuracy and speed. In the dataset used for the latent-factor analyses, the three executive-function components were moderately correlated: inhibitory control with working memory, r = 0.49; inhibitory control with cognitive flexibility, r = 0.45; and working memory with cognitive flexibility, r = 0.48.

**Figure 1:**
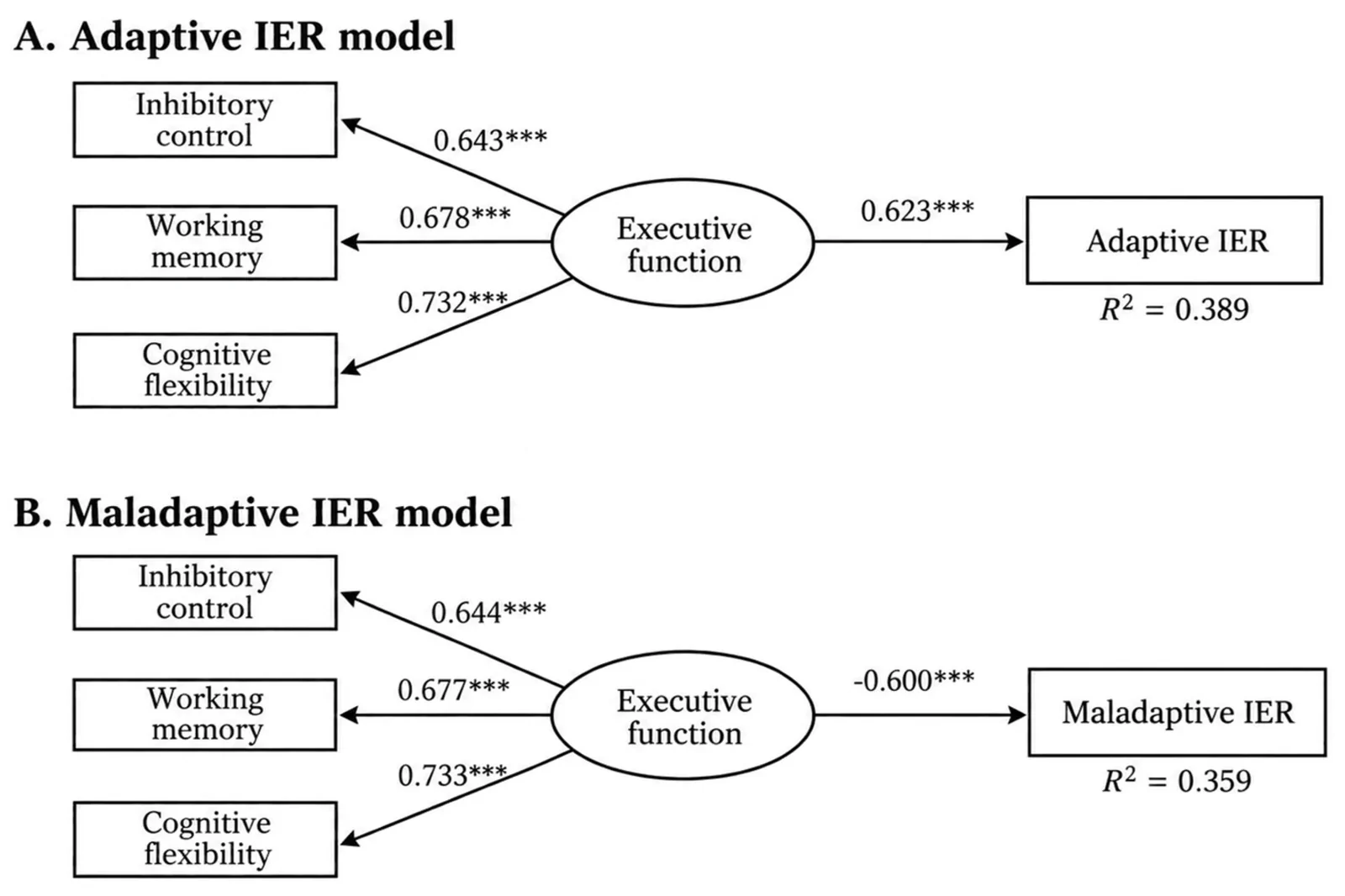
Latent executive-function models predicting adaptive and maladaptive IER. Note: Standardized coefficients are shown. *** *p* < 0.001.

The latent executive-function models are summarized in Figure 1 and Table 4, which present the model structure, fit statistics, standardized factor loadings, structural paths, and corresponding uncertainty estimates.

The latent executive-function models showed that the three component scores formed a coherent common factor. For adaptive IER, model fit was χ^2^(2) = 3.09, *p* = 0.213, CFI = 0.990, TLI = 0.971, RMSEA = 0.066, 90% CI [0.000, 0.202], and SRMR = 0.024. Standardized factor loadings were 0.643 (SE = 0.072, 95% Wald CI [0.502, 0.784]) for inhibitory control, 0.732 (SE = 0.066, 95% CI [0.603, 0.861]) for cognitive flexibility, and 0.678 (SE = 0.069, 95% CI [0.543, 0.813]) for working memory. Latent executive function was positively associated with adaptive IER, where β = 0.623, SE = 0.072, 95% CI [0.482, 0.764], *p* < 0.001, accounting for 38.9% of the variance in adaptive IER (R^2^ = 0.389).

For maladaptive IER, model fit was χ^2^(2) = 3.35, *p* = 0.187, CFI = 0.988, TLI = 0.964, RMSEA = 0.074, 90% CI [0.000, 0.207], and SRMR = 0.025. The corresponding standardized loadings were 0.644 (SE = 0.072, 95% CI [0.503, 0.785]) for inhibitory control, 0.733 (SE = 0.067, 95% CI [0.602, 0.864]) for cognitive flexibility, and 0.677 (SE = 0.070, 95% CI [0.540, 0.814]) for working memory. Latent executive function was negatively associated with maladaptive IER, with β = −0.600, SE = 0.075, 95% CI [−0.747, −0.453], *p* < 0.001, accounting for 35.9% of its variance (R^2^ = 0.359). The SEMs were estimated without age as an additional covariate.

Sensitivity analyses that additionally included sex in the component-level regression models retained the same FDR-based inference for five of the six associations. The working-memory association with maladaptive IER was attenuated, where B = −1.55, 95% CI [−3.17, 0.08], *p* = 0.062, q = 0.062. Thus, the main age-adjusted pattern was broadly robust, although this specific association was sensitive to additional adjustment for sex.

### 3.2. Study 2b: Social-Cognitive Correlates

#### 3.2.1. Materials and Methods

**Participants.** Study 2b used the same sample of 220 children described in Study 1. The original planning benchmark for the focal association analyses was based on a two-tailed test of a correlation coefficient with r = 0.30, α = 0.05, and power (1 − β) = 0.80, corresponding to a minimum sample size of 82. Because the full Study 1 sample was available for these analyses, the analyzed sample exceeded this original benchmark.

**Interpersonal emotion regulation.** The vignette-based IER assessment was identical to that used in Study 1. The same task-based adaptive and maladaptive summary indices served as the outcome variables.

**Empathy.** Empathy was assessed using the 20-item Basic Empathy Scale (BES; Jolliffe & Farrington, 2006), using the Chinese-language adaptation developed by Li et al. (2011). Cognitive empathy indexes the ability to identify and understand another person’s emotional state, whereas affective empathy reflects sensitivity and emotional responsiveness to another person’s affective experience (Reniers et al., 2011). The scale contains 20 items rated on a 5-point scale ranging from completely disagree to completely agree, with eight reverse-scored items. The Chinese adaptation was originally evaluated in Chinese adolescents; in the present sample of 8–10-year-old children, internal consistency was α = 0.86 for cognitive empathy and α = 0.81 for affective empathy.

**Theory of mind.** ToM was assessed using two second-order false-belief stories (Astington et al., 2002), with story order counterbalanced across participants. Second-order false-belief reasoning requires the child to infer one person’s belief about another person’s belief or knowledge. Each story included control questions followed by a second-order false-belief target question. Correct responses to each target question received one point and incorrect responses received zero points, yielding a total ToM score ranging from 0 to 2. The complete story materials are provided in the Supplementary Materials.

**Procedure.** Children were tested individually. The experimenter interacted with children in their classroom before testing to reduce unfamiliarity. The IER assessment was administered before the corresponding social-cognitive measure in each assessment sequence.

**Statistical analysis.** Data were managed using Microsoft Excel, and the social-cognitive analyses were conducted using Python 3.13.5 with statsmodels 0.14.6. Cognitive empathy, affective empathy, and theory of mind (ToM) were examined in separate ordinary least-squares regression models predicting adaptive and maladaptive IER, with exact age in months included as a continuous covariate. HC3 heteroscedasticity-robust standard errors were used for statistical inference. The six focal predictor–outcome coefficients were treated as a single family of tests and adjusted using the Benjamini–Hochberg false discovery rate (FDR) procedure. Unstandardized coefficients, 95% confidence intervals (CIs), standardized β coefficients, R^2^, and incremental ΔR^2^ values are reported. Pearson correlations described the bivariate associations among cognitive empathy, affective empathy, and ToM, with the three correlations adjusted using FDR; Spearman correlations were examined as a sensitivity check because ToM contained only three ordered score levels. ToM was entered as a 0–2 ordered linear-trend predictor in the primary models and as a three-level categorical predictor in sensitivity analyses. Sex was additionally included in sensitivity models.

#### 3.2.2. Results

Associations between social-cognitive capacities and IER are summarized in Table 5. Cognitive empathy was positively associated with adaptive IER after adjustment for exact age, where B = 0.71, 95% CI [0.46, 0.95], β = 0.349, R^2^ = 0.217, *p* < 0.001, FDR-adjusted q < 0.001, and negatively associated with maladaptive IER, with B = −0.21, 95% CI [−0.39, −0.03], β = −0.169, R^2^ = 0.059, *p* = 0.019, q = 0.019.

Affective empathy showed the strongest social-cognitive association with adaptive IER, where B = 0.88, 95% CI [0.70, 1.06], β = 0.546, R^2^ = 0.382, *p* < 0.001, q < 0.001. It was also negatively associated with maladaptive IER, where B = −0.21, 95% CI [−0.34, −0.08], β = −0.212, R^2^ = 0.074, *p* = 0.001, q = 0.002.

ToM was positively associated with adaptive IER, where B = 7.34 per one-point increase in ToM, 95% CI [5.51, 9.16], β = 0.468, R^2^ = 0.220, *p* < 0.001, q < 0.001, and negatively associated with maladaptive IER, where B = −2.08, 95% CI [−3.43, −0.74], β = −0.216, R^2^ = 0.050, *p* = 0.002, q = 0.003. ToM scores were distributed across all three levels (0: *n* = 71, 32.3%; 1: *n* = 85, 38.6%; 2: *n* = 64, 29.1%), with no pronounced concentration at the maximum score. Treating ToM as a three-level categorical predictor produced the same directional pattern for both IER outcomes.

The three social-cognitive measures were positively related to one another. Cognitive empathy correlated with affective empathy, r = 0.274, 95% CI [0.147, 0.392], q < 0.001; cognitive empathy correlated with ToM, r = 0.232, 95% CI [0.102, 0.353], q < 0.001; and affective empathy correlated with ToM, r = 0.393, 95% CI [0.275, 0.499], q < 0.001. Spearman correlations yielded the same pattern. Adding sex as a covariate did not change the direction or FDR-based inference of any of the six social-cognitive associations, and sex was not significant in these models.

Taken together, the findings from Study 2 supported H2. Better inhibitory control, working memory, and cognitive flexibility were associated with greater adaptive and lower maladaptive IER, and cognitive empathy, affective empathy, and ToM showed the same directional pattern. All six primary social-cognitive associations remained statistically supported after FDR correction, indicating systematic links between both executive and social-cognitive capacities and children’s reported regulatory tendencies.

## 4. Study 3: Contextual Modulation of Interpersonal Emotion Regulation

Study 3 examined whether children’s interpersonal emotion regulation varied systematically as a function of social context. Study 3a focused on regulatory goals, manipulating whether another child’s performance was aligned or in conflict with the participant’s own outcome in tasks for which different emotional states were instrumentally useful. Study 3b focused on regulatory demands, manipulating the urgency with which a distressed peer’s negative emotion needed to be down-regulated. The two studies used separate samples and provided complementary tests of context-sensitive interpersonal regulation.

### 4.1. Study 3a: Regulatory Goals

#### 4.1.1. Materials and Methods

**Participants.** The original a priori sample-size calculation was conducted using G*Power 3.1 (Faul et al., 2007) for a two-group between-participants comparison. Assuming an effect size of f = 0.40, α = 0.05, and power (1 − β) = 0.80, the calculation indicated a minimum total sample size of 52. This calculation served as the original recruitment benchmark. The final sample consisted of 252 children aged 8–10 years recruited from an urban elementary school. Children were randomly assigned to a cooperation condition (*n* = 126) or a competition condition (*n* = 126), with age and sex distributed as evenly as possible across conditions. All participating children were reported to be in good physical health and to have normal vision.

In the cooperation condition, the 8-year-old group included 47 children (24 boys and 23 girls; *M* age = 100.85 months, *SD* = 3.67), the 9-year-old group included 43 children (22 boys and 21 girls; *M* age = 113.07 months, *SD* = 3.54), and the 10-year-old group included 36 children (19 boys and 17 girls; *M* age = 125.36 months, *SD* = 3.17). In the competition condition, the corresponding groups included 50 children (27 boys and 23 girls; *M* age = 101.78 months, *SD* = 3.30), 39 children (21 boys and 18 girls; *M* age = 113.64 months, *SD* = 3.85), and 37 children (17 boys and 20 girls; *M* age = 124.78 months, *SD* = 4.18), respectively.

**Design and materials.** The study used a 2 (social goal: cooperation vs. competition; between participants) × 2 (game type: aggression-oriented vs. approach-oriented; within participants) mixed design. The primary outcome was children’s choice between happiness-inducing and anger-inducing recall material for another child before each game.

The regulatory-goal paradigm was adapted for children from prior instrumental emotion-regulation research, particularly Netzer et al. (2015). In their Study 3, adult participants were assigned to partnership or rivalry conditions and considered another person’s performance in shooting and dancing games, with anger and happiness, respectively, treated as potentially performance-facilitating emotions. The present study retained this instrumental structure while adapting the procedure for 8–10-year-old children and using a mixed design in which each child completed both game contexts. The aggression-oriented task was a single-player shooting game in which better performance involved defeating more opponents while being defeated fewer times; prior experimental work has shown that anger can improve performance in confrontational tasks, including a first-person shooting game (Tamir et al., 2008). The approach-oriented task was a dance game in which performance depended on accurately imitating movements presented on the screen; prior interpersonal instrumental-regulation research similarly treated happiness as useful for dancing performance and found that participants expected happiness to be more useful than anger or fear in that context (Netzer et al., 2015).

The emotion-induction component was informed by prior work showing that preferences for emotion-inducing activities vary with the instrumental demands of an upcoming goal (Tamir & Ford, 2009). In the present child adaptation, the happiness-induction material instructed the target to recall a personally experienced event that had made them happy, whereas the anger-induction material instructed the target to recall an event that had made them angry. Children additionally rated how much they wanted the target to experience happiness or anger, how much they wanted the target to complete the corresponding recall task before the game, and how useful they believed the relevant emotional state would be for the target’s game performance. The perceived-utility ratings were included to assess children’s own beliefs about whether the relevant emotion would facilitate the target’s performance, paralleling utility assessments used in prior interpersonal instrumental-regulation work (Netzer et al., 2015). These ratings were made on 7-point scales ranging from 1 (not at all) to 7 (very much).

**Procedure.** Children were told that they would participate in two online games with another participant and that their task was to select the recall material that the other participant would complete before each game. In the cooperation condition, the other child was described as a partner whose better performance would result in greater rewards for the participant as well. In the competition condition, the other child was described as an opponent whose better performance would result in fewer rewards for the participant. Children then selected either the happiness- or anger-inducing recall material for the target. After making their selection, they completed the ratings of desired target emotion, preference for the corresponding induction activity, and perceived emotional utility. No separate stand-alone comprehension check was administered for the cooperation/competition framing or for the instrumental usefulness of happiness and anger.

**Statistical analysis.** Data were managed using Microsoft Excel and analyzed in Python 3.13.5 using statsmodels 0.14.6. Because each child contributed a binary emotion-induction choice in both game conditions, choices were analyzed using binomial generalized estimating equations (GEE) with a logit link, with participant specified as the clustering variable, and an exchangeable working-correlation structure. Anger-inducing choices were coded 1 and happiness-inducing choices 0. Social goal (cooperation vs. competition), game type (aggression-oriented vs. approach-oriented), and their interaction were entered as predictors, with exact age in months included as a continuous covariate. Robust sandwich standard errors were used for inference. Significant interactions were followed by model-based simple contrasts with Holm adjustment for multiple comparisons; odds ratios (ORs) and 95% confidence intervals (CIs) are reported. Sensitivity analyses added sex as a covariate and estimated a participant-level random-intercept logistic mixed-effects model. Age-adjusted partial correlations examined associations among desired target emotion, preference for the corresponding induction activity, and perceived emotional utility, with the six correlations adjusted using the Benjamini–Hochberg false discovery rate procedure. These ratings were treated as secondary convergent measures rather than as manipulation or comprehension checks.

#### 4.1.2. Results

**Figure 2:**
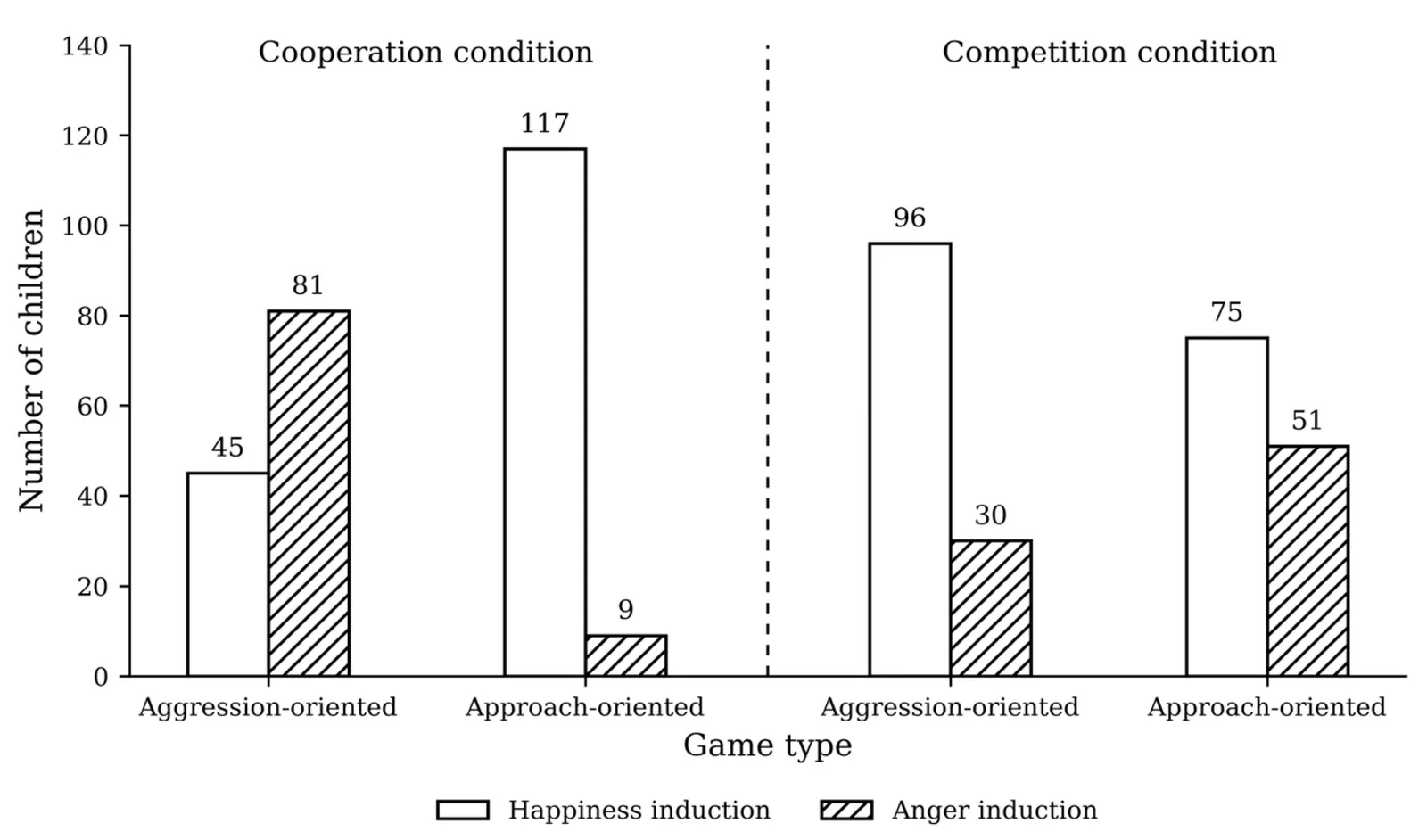
Regulatory-goal modulation of emotion-induction choices (Study 3a).

Emotion-induction choices varied as a joint function of social goal and game-type (Figure 2). The GEE model showed a significant social goal × game type interaction, where B = 3.93, robust SE = 0.51, Wald χ^2^(1) = 60.45, *p* < 0.001, OR = 50.95, 95% CI [18.92, 137.25]. Exact age was not significantly associated with choice, where B = −0.03 per 12 months, *p* = 0.803. In the aggression-oriented game, 81 children in the cooperation condition selected anger-inducing material and 45 selected happiness-inducing material, whereas the corresponding counts in the competition condition were 30 and 96. Competition therefore reduced the odds of selecting anger-inducing material relative to cooperation, where OR = 0.17, 95% CI [0.10, 0.30], Holm-adjusted *p* < 0.001.

In the approach-oriented game, 117 children in the cooperation condition selected happiness-inducing material and 9 selected anger-inducing material; in the competition condition, the corresponding counts were 75 and 51. Competition increased the odds of selecting anger-inducing material relative to cooperation, with OR = 8.85, 95% CI [4.11, 19.04], Holm-adjusted *p* < 0.001. The corresponding within-goal contrasts were also significant: anger-inducing choices were less likely in the approach-oriented than in the aggression-oriented game under cooperation, where OR = 0.04, 95% CI [0.02, 0.09], and Holm-adjusted *p* < 0.001, but more likely under competition, with OR = 2.18, 95% CI [1.19, 3.97], and Holm-adjusted *p* = 0.011. Thus, competition produced a substantial shift toward anger in the approach-oriented game, although happiness-inducing material remained the more frequent choice within that condition (75 vs. 51).

Children’s beliefs about the instrumental usefulness of emotion were also associated with their regulatory preferences. In the aggression-oriented game, while controlling for exact age, desired target emotion was positively associated with preference for the corresponding emotion-induction activity, where partial r = 0.415, 95% CI [0.307, 0.513], FDR-adjusted q < 0.001. Perceived emotional utility was positively associated with both desired target emotion, with partial r = 0.181, 95% CI [0.058, 0.298], q = 0.006, and preference for the corresponding induction activity, where partial r = 0.180, 95% CI [0.058, 0.298], q = 0.006.

A similar pattern was observed in the approach-oriented game. Desired target emotion was positively associated with preference for the corresponding emotion-induction activity, where partial r = 0.414, 95% CI [0.306, 0.512], FDR-adjusted q < 0.001. Perceived emotional utility was positively associated with desired target emotion, where partial r = 0.147, 95% CI [0.023, 0.266], q = 0.024, and with preference for the corresponding induction activity, where partial r = 0.141, 95% CI [0.017, 0.260], q = 0.025.

The social goal × game-type interaction was unchanged when sex was added as a covariate, with B = 3.93, OR = 51.00, 95% CI [18.92, 137.44], *p* < 0.001; sex was not associated with choice, *p* = 0.822. A participant-level random-intercept logistic mixed-effects sensitivity model produced virtually identical fixed-effect estimates, with the random-intercept variance approaching the boundary. Together, the results supported H3a by showing strong context-dependent modulation of emotion-induction choices in the predicted instrumental direction, while also indicating that instrumental considerations did not produce a complete reversal of emotional preference in every experimental cell.

### 4.2. Study 3b: Regulatory Demands

#### 4.2.1. Materials and Methods

**Participants.** The original a priori sample-size calculation was conducted using G*Power 3.1 (Faul et al., 2007) for an F test of the repeated-measures within–between interaction. The calculation assumed f = 0.25, α = 0.05, power (1 − β) = 0.80, two groups, four repeated measurements, a correlation of 0.50 among repeated measures, and a nonsphericity correction of ε = 1, yielding a minimum total sample size of 48. This calculation reflected the original 2 × 4 mixed-design analysis framework. The final sample consisted of 250 children aged 8–10 years recruited from an urban elementary school. Children were randomly assigned to a high-demand condition (*n* = 125) or a low-demand condition (*n* = 125). The high-demand group included 65 boys and 60 girls (*M* age = 112.08 months, *SD* = 10.18), whereas the low-demand group included 68 boys and 57 girls (*M* age = 111.94 months, *SD* = 10.32). All participating children were reported to be in good physical health and to have normal vision.

**Regulatory-demand manipulation and IER assessment.** Regulatory demand was manipulated using a modified vignette-based paradigm adapted from Pauw et al. (2019) and the Study 1 scenario. In the low-demand condition, the target had reached the end of the school day after the stressful classroom event and had ample time before any further tasks. In the high-demand condition, the same event was followed by an imminent examination. The examination context was intended to increase the temporal urgency of down-regulating the target’s negative emotion by introducing an immediate competing task. No separate direct rating of perceived urgency or regulatory demand was administered.

After the vignette, children rated concrete interpersonal regulatory responses representing cognitive engagement, affective engagement, distraction, and suppression. As in Study 1, ratings were made at the response-item level rather than by asking children to classify abstract strategy labels. Children rated the extent to which they would use each response on a 7-point Likert-type scale ranging from 1 (not at all) to 7 (very much). Item ratings corresponding to each strategy were aggregated to obtain a strategy-level score, with higher scores indicating greater endorsement of that strategy. Study 3b therefore retained the item-level response format of the Study 1 vignette assessment while analyzing the four strategy scores as distinct outcomes.

**Statistical analysis.** Data were managed using Microsoft Excel, and the regulatory-demand analyses were conducted using Python 3.13.5 with statsmodels 0.14.6. Because cognitive engagement, affective engagement, distraction, and suppression represented distinct regulatory-strategy outcomes rather than levels of an experimentally manipulated within-participant factor, each strategy score was analyzed separately using ordinary least-squares regression. Regulatory demand was coded 1 for the high-demand condition and 0 for the low-demand condition, and exact age in months was included as a continuous covariate. HC3 heteroscedasticity-robust standard errors were used for statistical inference. The four regulatory-demand coefficients were treated as a single family of tests and adjusted using the Benjamini–Hochberg false discovery rate (FDR) procedure. Effect estimates are reported as adjusted high-minus-low differences with 95% confidence intervals (CIs), standardized β coefficients, and R^2^ values. A sensitivity analysis additionally included sex as a covariate. The suppression model was estimated using the full Study 3b sample (N = 250).

#### 4.2.2. Results

**Figure 3:**
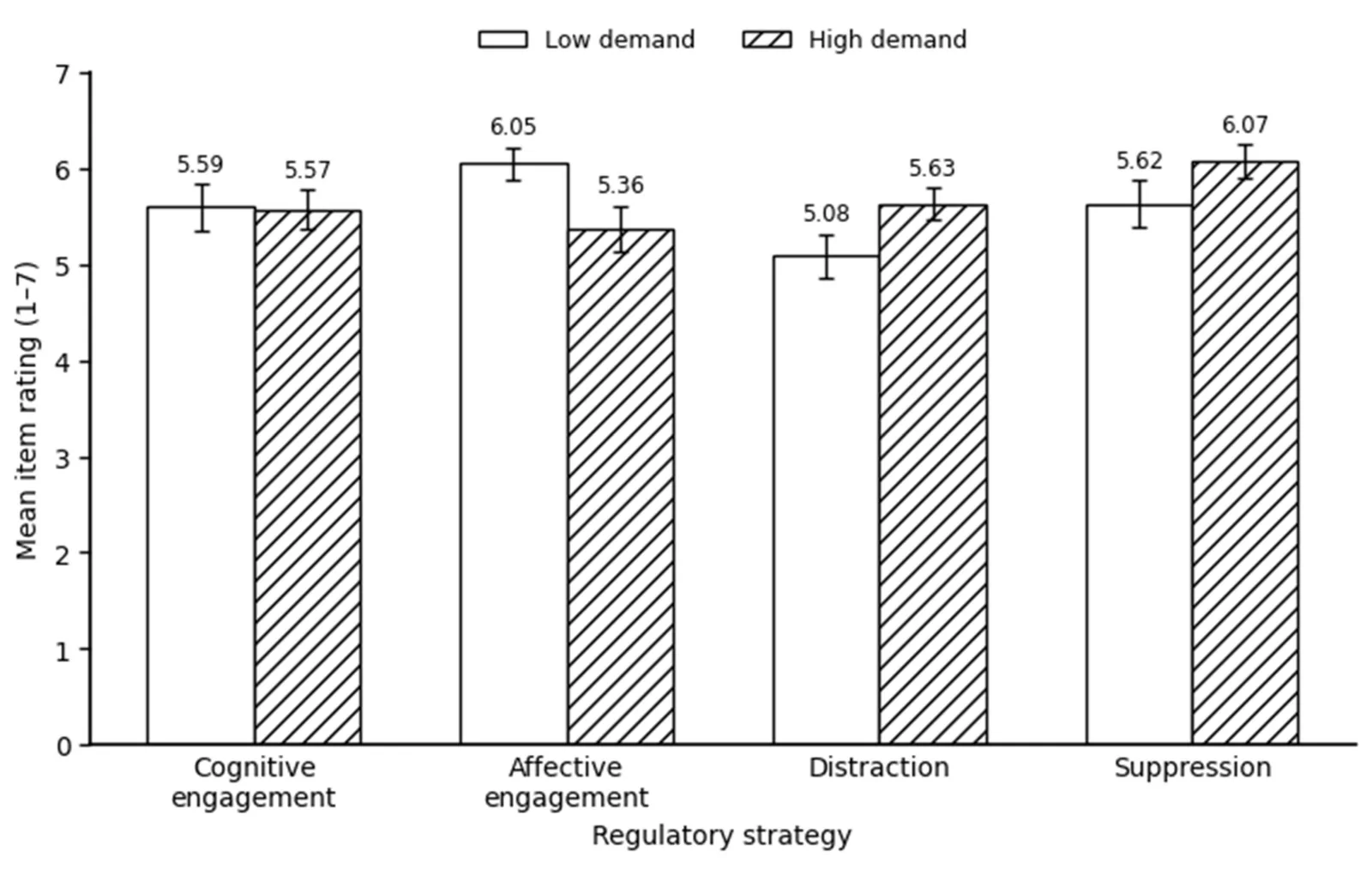
Strategy scores across regulatory-demand conditions.

Strategy scores across regulatory-demand conditions are presented in Figure 3.

Cognitive engagement did not differ significantly between the high- and low-demand conditions after adjustment for exact age, whereby B = −0.10, 95% CI [−1.34, 1.13], β = −0.010, R^2^ = 0.013, *p* = 0.870, FDR-adjusted q = 0.870. In contrast, children in the high-demand condition reported lower affective engagement, with B = −2.75, 95% CI [−3.93, −1.57], β = −0.279, R^2^ = 0.089, *p* < 0.001, q < 0.001.

High regulatory demand was also associated with greater distraction, where B = 2.17, 95% CI [1.00, 3.34], β = 0.226, R^2^ = 0.055, *p* < 0.001, q < 0.001, and greater suppression, where B = 0.88, 95% CI [0.28, 1.49], β = 0.179, R^2^ = 0.039, *p* = 0.004, q = 0.006. These coefficients represent the adjusted difference between the high- and low-demand conditions.

Adding sex as a covariate did not change the direction or FDR-based inference of any regulatory-demand effect. Accordingly, H3b was partially supported. Higher regulatory demand was associated with lower affective engagement and greater distraction and suppression, whereas the predicted reduction in cognitive engagement was not observed.

Taken together, Studies 3a and 3b indicate that children’s interpersonal regulatory responses varied across two distinct contextual manipulations: the instrumental consequences of the target’s emotional state for an ongoing social goal and the presence versus absence of an imminent examination intended to increase regulatory urgency. These findings provide the contextual basis for Study 4, which examines whether general cognitive-control capacity and social cognition are associated with variation in this context sensitivity.

## 5. Study 4: Individual Differences in Context Sensitivity

Study 4 examined whether children’s responsiveness to interpersonal context varied as a function of broader cognitive and social-cognitive capacities. General cognitive-control capacity was represented by a composite of inhibitory control, working memory, and cognitive flexibility, whereas social cognition was represented by a composite of empathy and theory of mind (ToM). We tested whether these two capacities moderated children’s responses to regulatory goals and regulatory demands. These analyses extend Studies 2 and 3 by asking not only whether individual capacities and contextual features are associated with IER, but whether broader domains of capacity are related to the extent to which children’s regulatory choices vary across interpersonal contexts.

### 5.1. Materials and Methods

#### 5.1.1. Participants

Study 4 included 125 children aged 8–10 years (M age = 111.60 months, SD = 10.36, range = 96–130 months), who were randomly selected from the pool of participants who had completed the full set of measures and task paradigms required for the person-by-context analyses, including the executive-function, empathy, ToM, regulatory-goal, and regulatory-demand assessments. Study 4 therefore represents an integrative analysis of an overlapping participant subset rather than a separately recruited cohort. No separate a priori power analysis was conducted specifically for the Study 4 moderation hypotheses.

For both contextual paradigms, 30 children contributed observations under both levels of the contextual manipulation, whereas the remaining 95 children contributed observations under one contextual level. The regulatory-goal dataset therefore comprised 155 child-by-goal observations (78 cooperation and 77 competition), and the regulatory-demand dataset comprised 155 child-by-demand observations (78 low demand and 77 high demand). Repeated observations contributed by the same child were accounted for in the statistical models.

#### 5.1.2. Individual Difference and Contextual Measures

Executive function was assessed using the inhibitory-control, working-memory, and cognitive-flexibility tasks described in Study 2a. The three component scores were summed and standardized to index general cognitive-control capacity, with higher scores indicating better overall cognitive-control performance. Empathy and ToM were assessed using the measures described in Study 2b. The empathy total score and the original ToM score were separately standardized, averaged with equal weight, and standardized again to form the social-cognitive composite, with higher scores indicating greater social-cognitive capacity. Empathy and ToM were moderately positively associated in the Study 4 sample, r = 0.38.

Regulatory goals were assessed using the same cooperation-versus-competition paradigm described in Study 3a. Children selected happiness- or anger-inducing recall material for another child before the aggression-oriented and approach-oriented games. Regulatory demand was assessed using the high- versus low-demand vignette paradigm described in Study 3b. In these analyses, the outcomes were children’s ratings of cognitive engagement, affective engagement, distraction, and suppression.

#### 5.1.3. Statistical Analysis

For Study 4, individual differences were represented by two continuous capacity indices. Inhibitory control, working memory, and cognitive flexibility component scores were combined to index general cognitive-control capacity. Empathy and ToM scores were standardized and combined with equal weighting to form a social-cognitive composite. These indices were used to capture broader individual-capacity domains relevant to the person × context hypotheses.

Because the Study 4 data included repeated observations within participants, regulatory-goal choices were analyzed using participant-clustered binomial generalized estimating equations (GEE), whereas regulatory-strategy outcomes were analyzed using participant-clustered Gaussian GEE. For the regulatory-goal analyses, each continuous capacity index was entered as a moderator together with regulatory goal, game type, and their interaction terms. For the regulatory-demand analyses, each capacity index, regulatory demand, and their interaction were entered as predictors of the strategy outcomes.

Data were managed using Microsoft Excel and analyzed in Python 3.13.5 using statsmodels 0.14.6. Continuous individual-difference composites were standardized before analysis, and exact age in months was included as a continuous covariate. For regulatory-goal analyses, the binary emotion-induction choices were analyzed using binomial generalized estimating equations (GEE) with a logit link, participant specified as the clustering variable, and an exchangeable working-correlation structure. Anger-inducing choices were coded 1 and happiness-inducing choices 0, consistent with Study 3a. Separate models were estimated for general cognitive-control capacity and social cognition. Each model included regulatory goal, game type, the focal individual-difference composite, all two-way interactions, and the three-way interaction among capacity, regulatory goal, and game type. The primary moderation test was a two-degree-of-freedom joint Wald test of the capacity × regulatory goal and capacity × regulatory goal × game-type terms. The two primary omnibus moderation tests were adjusted using the Benjamini–Hochberg false discovery rate (FDR) procedure. Significant omnibus effects were followed by game-specific capacity × regulatory-goal contrasts with Holm adjustment. For regulatory-demand analyses, cognitive engagement, affective engagement, distraction, and suppression were analyzed separately using Gaussian GEE models with participant-level clustering. Each model included regulatory demand, one of the two continuous capacity composites, their interaction, and exact age in months. The eight prespecified capacity × demand interaction tests (two capacities × four outcomes) were treated as one inferential family and adjusted using Benjamini–Hochberg FDR. Significant interactions were decomposed using model-based simple effects at −1 SD, the mean, and +1 SD of the moderator. One suppression score exceeded the possible 2–14 range and was treated as missing for that outcome.

### 5.2. Results

#### 5.2.1. Individual Differences in Responsiveness to Regulatory Goals

Interactions between individual capacities and regulatory goals are summarized in Table 6. General cognitive-control capacity significantly moderated children’s responsiveness to regulatory goal across the two game contexts, with Wald χ^2^(2) = 7.45, *p* = 0.024. The effect remained significant after FDR correction across the two primary regulatory-goal moderation tests, where q = 0.048. The general cognitive control × regulatory goal × game-type interaction itself was not significant, where B = −0.97, SE = 0.88, 95% CI [−2.69, 0.76], *p* = 0.272, indicating that the difference between the two game-specific moderation effects was not statistically reliable.

Follow-up contrasts indicated that general cognitive-control capacity moderated regulatory-goal-related choices in the approach-oriented game, with B = −1.48, SE = 0.66, OR = 0.23, 95% CI [0.06, 0.83], *p* = 0.025, Holm-adjusted *p* = 0.049. The corresponding interaction in the aggression-oriented game was not significant, where B = −0.51, SE = 0.47, OR = 0.60, 95% CI [0.24, 1.52], *p* = 0.282. In contrast, social cognition did not significantly moderate regulatory-goal responsiveness, where Wald χ^2^(2) = 0.01, *p* = 0.997, q = 0.997. The social cognition × regulatory goal interaction was nonsignificant in both the aggression-oriented and approach-oriented games (Holm-adjusted ps = 1.00).

Taken together, general cognitive-control capacity was associated with variation in children’s regulatory-goal responsiveness, with the clearest game-specific association emerging in the approach-oriented context. Social cognition was not associated with regulatory-goal responsiveness.

#### 5.2.2. Individual Differences in Responsiveness to Regulatory Demands

Interactions between individual capacities and regulatory demand across IER strategies are summarized in Table 7. General cognitive-control capacity did not significantly moderate the effect of regulatory demand on cognitive engagement, affective engagement, distraction, or suppression after correction for multiple testing (all qs ≥ 0.536).

Social cognition showed a selective interaction with regulatory-demand. The social cognition × regulatory demand interaction was significant for cognitive engagement, where B = −1.97, SE = 0.70, 95% CI [−3.35, −0.60], Wald χ^2^(1) = 7.93, *p* = 0.0049. This interaction remained significant following FDR correction across the eight prespecified capacity × demand tests, where q = 0.039.

Model-based simple-effect analyses showed that, among children with relatively lower social-cognitive scores (−1 SD), high regulatory demand was associated with greater cognitive engagement than low regulatory demand, given that B = 2.68, SE = 1.10, 95% CI [0.54, 4.83], *p* = 0.014, Holm-adjusted *p* = 0.043. The high-versus-low demand contrast was not significant at the mean level of social cognition, where B = 0.71, SE = 0.58, 95% CI [−0.42, 1.84], *p* = 0.220, or at relatively high social cognition (+1 SD), with B = −1.27, SE = 0.67, 95% CI [−2.58, 0.05], *p* = 0.059, Holm-adjusted *p* = 0.118. Social cognition did not significantly moderate regulatory-demand effects on affective engagement, distraction, or suppression after FDR correction.

#### 5.2.3. Hypothesis Evaluation

Overall, H4 received selective support. General cognitive-control capacity moderated responsiveness to regulatory goals, with the clearest game-specific association emerging in the approach-oriented context. Social cognition did not moderate regulatory-goal-related emotion-induction choices. For regulatory demand, social cognition moderated cognitive engagement, whereas general cognitive-control capacity did not significantly moderate any of the four strategy outcomes after correction for multiple testing.

These findings indicate that person-by-context effects in children’s IER were domain-specific rather than uniform. General cognitive-control capacity was associated primarily with responsiveness to regulatory goals, whereas social cognition was associated with demand-related variation in cognitive engagement.

## 6. General Discussion

The present research examined interpersonal emotion regulation (IER) in middle childhood by integrating age-related variation, individual cognitive capacities, contextual modulation, and person-by-context differences within a common framework. Across four linked studies, the findings converge on the view that children’s regulation of others’ emotions cannot be characterized solely in terms of greater or lesser use of particular strategies. Instead, IER appears to involve both individual capacities that support regulatory decisions and sensitivity to the interpersonal circumstances in which those decisions are made. Study 1 showed age-related differences in several adaptive and maladaptive IER strategies. Study 2 demonstrated systematic associations between IER and both executive and social-cognitive capacities. Study 3 showed that children altered their regulatory choices as social goals and regulatory demands changed. Finally, Study 4 showed selective person-by-context effects: general cognitive-control capacity moderated responsiveness to regulatory goals, whereas social cognition moderated demand-related variation in cognitive engagement. Together, the findings support a progression from age-related variation, to cognitive capacity, to contextual modulation, and ultimately to individual differences in context sensitivity.

### 6.1. Age-Related Patterns in Interpersonal Emotion Regulation

Study 1 provides evidence of systematic age-related variation in children’s reported interpersonal regulatory tendencies across the observed 8–10-year range. Higher age was associated with greater cognitive and affective engagement and with lower avoidance and co-rumination. Distraction and suppression were not significantly associated with age. The secondary composite analyses showed the same overall pattern, with higher adaptive IER and lower maladaptive IER at older ages. These associations were robust to adjustment for sex, and there was no evidence that quadratic age terms provided a better account of the data. Accordingly, H1 was partially supported.

The contrast between the positive age-related associations for engagement strategies and the absence of a significant age association for distraction is theoretically informative. Cognitive and affective engagement require the regulator to remain oriented toward the target’s experience, represent aspects of the stressful event, and generate a response that addresses either the target’s interpretation or emotional state. These responses may therefore place greater demands on the developing capacity to understand another person’s perspective and formulate an interpersonal response. By contrast, distraction provides a relatively direct route for redirecting attention away from distress and may require a less-elaborated representation of the target’s emotional experience. This pattern is broadly consistent with previous developmental work showing increasingly elaborated interpersonal regulatory responses across childhood (López-Pérez et al., 2016; López-Pérez & Pacella, 2021).

At the same time, these results should not be interpreted as evidence for a uniform developmental shift from “maladaptive” to “adaptive” regulation. Suppression, in particular, did not show the predicted decrease. This null finding becomes especially important when considered alongside Study 3, in which suppression increased under high regulatory demand. Thus, although the adaptive and maladaptive labels remain useful for describing the measurement framework employed in Studies 1 and 2, they should not be treated as immutable functional properties of the strategies themselves. A strategy that is generally classified as maladaptive may nevertheless be selected more often when particular situational demands make immediate disengagement from an emotional experience more salient. This distinction helps connect the age-related findings with regulatory-flexibility perspectives emphasizing strategy–situation fit rather than universally optimal strategies (Bonanno & Burton, 2013; Doré et al., 2016).

Because Study 1 was cross-sectional, the observed associations indicate age-related variation rather than within-child developmental change. More importantly, for the theoretical argument of this article, the pattern suggests increasing differentiation in children’s interpersonal regulatory responses rather than a uniform movement toward one globally optimal strategy profile. These cross-sectional findings are consistent with the possibility that IER development involves an expanding capacity to deploy more elaborated responses while preserving strategies whose usefulness depends on situational demands.

### 6.2. Cognitive and Social-Cognitive Correlates of Interpersonal Emotion Regulation

Study 2 addressed the capacities that may support these interpersonal regulatory tendencies. Consistent with H2, inhibitory control, working memory, and cognitive flexibility were each positively associated with adaptive IER and negatively associated with maladaptive IER in the primary regressions controlling for exact age. Cognitive flexibility showed the largest standardized component-level associations. Latent executive-function models, estimated without an additional age covariate, showed the same directional pattern: the common EF factor was positively associated with adaptive IER and negatively associated with maladaptive IER. Sensitivity analyses additionally controlling for sex left five of the six component-level associations statistically supported, whereas the working-memory association with maladaptive IER was attenuated. In the social-cognitive analyses, cognitive empathy, affective empathy, and ToM were each positively associated with adaptive IER and negatively associated with maladaptive IER after adjustment for exact age and FDR correction. Affective empathy showed the strongest standardized association with adaptive IER, and the social-cognitive findings were unchanged when sex was added as a covariate.

These findings are consistent with the idea that IER places demands on at least two complementary forms of capacity. Executive functions may contribute to the control and coordination of regulation. Inhibitory control can support the suppression of a dominant but ineffective response; working memory can help maintain goal-relevant information while alternative responses are considered; and cognitive flexibility can support adjustment among possible regulatory responses. These functions map naturally onto process accounts in which regulators must represent a target state, maintain a regulatory objective, evaluate alternative responses, and select an action (Reeck et al., 2016; Diamond, 2013; Hudson & Jacques, 2014; Simonds et al., 2007). The present task scores should be interpreted as broad performance composites that integrate accuracy and speed. In particular, the cognitive-flexibility measure reflects performance in the mixed Flanker condition, which required children to alternate between the standard and reversed response rules, rather than an isolated trial-level switch-cost parameter.

Empathy and ToM, by contrast, may contribute more directly to the representation of the target. Regulating another person requires more than controlling one’s own response: the regulator must infer what the other person is experiencing and, in many situations, what the person believes, intends, or needs. Empathy may facilitate sensitivity to the target’s emotional experience, whereas ToM may support reasoning about mental states and perspectives (Zaki, 2020). The associations observed in Study 2 therefore suggest that effective IER may depend jointly on the capacity to control regulatory processing and the capacity to represent the person whose emotion is being regulated.

These results should remain associative rather than causal in interpretation. Stronger executive or social-cognitive capacities were related to children’s reported IER tendencies, but the present data do not establish that these capacities cause particular strategies to develop. Nor do the measures identify a unique cognitive capacity with a unique temporal stage of the regulatory cycle. The theoretical return is therefore more specific: the findings are consistent with process accounts of IER by supporting a complementary-capacities view, in which executive functions may contribute to coordinating regulation whereas empathy and ToM may contribute to representing the target. Study 4 further indicates that these capacities are relevant not only to average IER tendencies but, selectively, to how regulation changes with interpersonal context.

### 6.3. Contextual Modulation and Context Sensitivity

Study 3 shifted the focus from what children were generally capable of doing to whether their regulatory decisions changed with the social situation. The findings from the regulatory-goal and regulatory-demand paradigms provide complementary evidence that IER in middle childhood is context sensitive.

In Study 3a, emotion-induction choices depended jointly on social goal and game-type. The robust social goal × game type interaction showed that cooperative and competitive goals shifted children’s choices in opposite directions across tasks with different instrumental emotion profiles. In the aggression-oriented game, cooperation was associated with more anger-inducing choices and competition with more happiness-inducing choices. In the approach-oriented game, cooperation strongly favored happiness-inducing material, whereas competition produced a substantial shift toward anger. The accompanying ratings of desired emotion, induction preference, and perceived emotional utility were consistent with the interpretation that children’s regulatory choices were sensitive to the perceived performance value of the relevant emotion.

This pattern is consistent with motivational accounts distinguishing hedonic from instrumental goals (Tamir, 2016; Netzer et al., 2015). Children did not simply attempt to make another person feel better. Rather, their choices varied according to what the target’s emotional state could accomplish within the broader interaction. When the target’s good performance benefited the participant, children tended to induce the emotion presented as useful for that performance. When the target’s performance conflicted with the participant’s own outcome, choices shifted in the opposite direction.

The evidence for H3a was nevertheless substantial rather than complete. In the competition condition of the approach-oriented game, competition significantly shifted choices toward anger relative to cooperation, but happiness-inducing material remained numerically more common than anger-inducing material within the competition group itself. Thus, the results demonstrate contextual modulation in the predicted instrumental direction without supporting a complete reversal of emotional preference in every condition. This distinction is theoretically useful: instrumental considerations influenced children’s regulatory decisions, but they did not necessarily eliminate other motives, including the general tendency to favor a positive emotional state.

Study 3b demonstrated a different form of contextual modulation. In the high-demand vignette, the distressed peer was described as facing an imminent examination, whereas no immediate task followed the stressful event in the low-demand condition. After adjustment for exact age and FDR correction, children in the high-demand condition reported greater distraction and suppression and lower affective engagement; cognitive engagement did not differ between conditions. H3b was therefore partially supported.

This condition-related pattern is consistent with the idea that strategy selection depends on the temporal and functional demands of regulation. Distraction can redirect attention relatively quickly, and suppression can restrict emotional expression, whereas affective engagement keeps both regulator and target more directly engaged with the emotional experience. The greater distraction and suppression observed in the high-demand condition are therefore consistent with sensitivity to an immediate regulatory problem rather than a simple preference for strategies conventionally categorized as adaptive. Similar arguments underlie regulatory-flexibility accounts and the recent work emphasizing that the consequences and usefulness of IER strategies depend on what regulation is intended to achieve (Pauw et al., 2019, 2026). Because perceived urgency was not measured independently, however, this interpretation concerns the intended contextual contrast rather than a directly verified subjective demand state. This distinction is consistent with the vignette-method guidance emphasizing the direct evaluation of manipulation success when feasible (Aguinis & Bradley, 2014; Hillen et al., 2025); future work could add a brief age-appropriate urgency rating alongside the focal IER outcomes.

Taken together, the two manipulations clarify two distinct functions of context in IER: regulatory goals changed the desired emotional endpoint, whereas regulatory demands changed the preferred route for reaching a regulatory endpoint. In this sense, the present findings extend context-sensitive accounts of emotion regulation from the question of which strategy is generally beneficial to the interpersonal question of what emotional state should be produced in another person, and by what means, under different social circumstances. They support the broader proposition that IER cannot be evaluated independently of regulatory goals and situational demands.

Importantly, the present findings provide evidence for context sensitivity, not for the entirety of regulatory flexibility. The studies manipulated contextual characteristics and showed systematic differences in children’s regulatory choices, but they did not examine real-time switching between strategies, monitoring of regulatory success, or adjustment following feedback. Context sensitivity should therefore be understood here as one component of a broader flexibility construct.

### 6.4. Individual Differences in Context Sensitivity

Study 4 provided the strongest test of the proposed integration between capacity and context. H4 predicted that general cognitive-control capacity and social cognition would interact with regulatory goals and demands, without specifying the direction of these interactions. The findings provided selective support.

For regulatory goals, general cognitive-control capacity significantly moderated children’s responsiveness after correction for multiple testing. The game-specific pattern was clearest in the approach-oriented task, whereas the corresponding interaction in the aggression-oriented task was not significant. The capacity × goal × game-type interaction was itself nonsignificant, indicating that the difference between the two game-specific moderation effects was not statistically reliable. Social cognition did not moderate regulatory-goal-related choices in either game context.

This pattern is consistent with the possibility that cognitive control becomes especially relevant when a child must maintain and coordinate several contingencies at once: which emotion is useful for the task, whether better target performance helps or harms the regulator, and what emotional outcome should therefore be produced. The general cognitive-control capacity measure integrates inhibitory control, working memory, and cognitive flexibility, capacities that may jointly support maintaining a context-specific objective while suppressing competing responses and updating regulatory decisions as interpersonal contingencies change.

The regulatory-demand findings showed a complementary pattern. General cognitive-control capacity did not significantly moderate cognitive engagement, affective engagement, distraction, or suppression after FDR correction. Social cognition, by contrast, moderated cognitive engagement: high regulatory demand was associated with greater cognitive engagement, primarily among children with relatively lower social-cognitive scores, whereas the high-versus-low demand difference diminished as social cognition increased. Social cognition did not significantly moderate the remaining strategy outcomes.

The difference between the goal and demand findings may reflect the distinct information-processing requirements of the two contextual dimensions. Regulatory-goal decisions explicitly required the coordination of task utility and cooperative or competitive consequences, which may place particular demands on cognitive control. Regulatory demand instead altered the urgency of responding to a distressed peer. In that setting, social-cognitive understanding may shape how strongly children increase cognitively engaging responses when the situation becomes more pressing. These interpretations concern selective associations rather than a general advantage of higher capacity across all contexts.

The theoretical implication is that context sensitivity should not be treated as a unitary individual-difference property. Sensitivity to regulatory goals and sensitivity to regulatory demands may draw on partly different capacities, and the same capacity need not predict adaptation to every contextual cue or every regulatory response. This refines a general person–situation account of emotion regulation: rather than asking whether a child is globally “flexible,” it may be more informative to ask which capacity supports sensitivity to which contextual feature and for which regulatory decision.

### 6.5. An Integrative Developmental Model of Child-to-Peer IER

**Figure 4:**
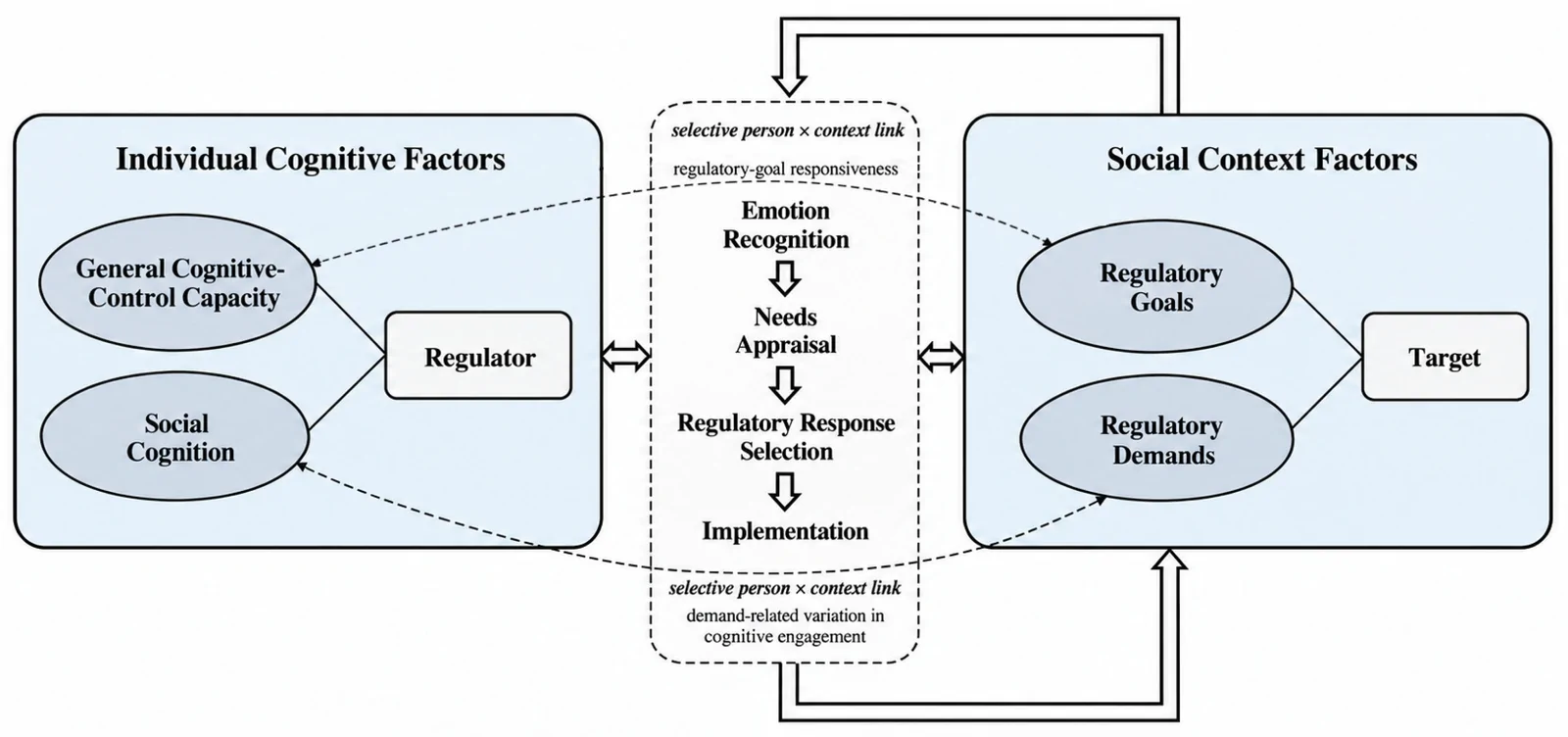
Integrative developmental model of child-to-peer interpersonal emotion regulation.

Building on the interpersonal emotion-regulation cycle model (Reeck et al., 2016), the present findings support an integrative developmental model of child-to-peer IER that brings together regulator-level capacities and social-context inputs within the same cyclical process (Figure 4). In this model, the regulatory sequence remains identifying the target’s emotion, appraising regulatory need, selecting a regulatory response, and implementing that response. What is added is a specification of the major classes of influences examined across the present studies.

On the regulator side, individual cognition comprises two broad classes of capacity: general cognitive-control capacity and social cognition. General cognitive-control capacity refers to the executive resources involved in maintaining goals, inhibiting prepotent responses, and flexibly coordinating alternatives, whereas social cognition refers to the child’s capacity to understand and represent the target’s emotional and mental states. On the social-context side, the target and situation provide cues about the regulatory goal and the regulatory demand. These contextual inputs shape what emotional outcome is sought and how urgently or directly the target’s state needs to be changed. The cyclical arrows in Figure 4 indicate that children’s IER is not a one-shot choice; implementation is embedded in an ongoing interpersonal exchange in which the target’s state and the interpersonal situation continue to provide feedback.

The present results further suggest that person and context influences are related but not interchangeable. Study 4 showed that general cognitive-control capacity was most clearly implicated in sensitivity to regulatory goals, whereas social cognition was most clearly implicated in demand-related variation in cognitive engagement. Accordingly, the model does not assume a single global flexibility factor. Rather, different capacities may be recruited at different points in the interpersonal regulation cycle and may support sensitivity to different contextual features. This formulation helps link developmental, cognitive, and contextual findings within a single account of how children regulate the emotions of peers.

### 6.6. Limitations and Future Directions

Several limitations define the scope of the present findings. First, the age-related analyses were cross-sectional. They therefore identify between-child associations with age rather than within-child developmental change. Longitudinal studies are needed to determine how changes in executive and social-cognitive capacities relate to changes in IER within children over time.

Second, the vignette-based measures captured children’s reported regulatory tendencies under controlled interpersonal conditions rather than enacted regulation in ongoing interactions. A broader developmental perspective places emotion regulation within reciprocal dyadic and co-regulatory processes rather than solely within capacities of the individual child. Work on parent–child dyads highlights coordinated attentional, neural, and physiological processes as part of emotional development (Christou & Bacopoulou, 2025), and real-time observations of parent–child emotion discussions demonstrate reciprocal emotion-related responding across interaction partners (Morelen & Suveg, 2012). Complementing this work, parent–child eye-tracking data show congruence in visual attention to emotional cues, including associations between parental and child gaze to angry eyes (Christou et al., 2025). Although this literature primarily concerns parent–child rather than peer dyads, it provides a broader developmental rationale for treating child-to-peer IER as an unfolding interpersonal process. Future work using live or repeated peer dyadic interactions could examine how children respond to target feedback, coordinate attention and affect, maintain or change regulatory strategies, and adapt across successive interpersonal exchanges. Such designs would also allow context sensitivity to be extended to other components of regulatory flexibility, including strategy switching, monitoring, and feedback-based adjustment.

Third, regulatory goals and regulatory demands were examined using different outcome formats—emotion-induction choices and strategy ratings, respectively. The present studies therefore do not provide a common metric for directly comparing sensitivity across contextual dimensions. In Study 3b, the regulatory-demand manipulation also did not include an independent direct measure of perceived urgency; the observed strategy differences therefore establish condition-related variation but do not by themselves verify that children experienced the high-demand vignette as more urgent. Study 4 represented broader individual capacities using composite indicators. Although this approach provides a parsimonious test of domain-level person-by-context effects, the social-cognitive composite should not be interpreted as implying that empathy and ToM are interchangeable or contribute equally to contextual sensitivity. Future work using broader multi-indicator measures could examine these higher-order capacities within latent-variable models. Because some analyses drew on the same participant samples, convergence across those analyses should not be interpreted as independent replication.

Relatedly, the vignette procedures can be situated more explicitly within established methodological guidance. Across the studies, the scenarios were grounded in prior paradigms, adapted to concrete child-to-peer contexts, reported with their sources and procedural details, and administered in samples that exceeded the original recruitment benchmarks. Study 1 also showed that most children identified the target emotion as intended. At the same time, the original studies did not include a separate formal pilot specifically evaluating realism/representativeness or a structured expert-rating stage, and the contextual manipulations were not accompanied by stand-alone manipulation checks in every study. In Study 3a, the desired-emotion, induction-preference, and perceived-utility ratings are therefore best viewed as convergent measures; in Study 3b, perceived urgency was not measured directly. Following the dimensions emphasized by Aguinis and Bradley (2014) and, as a complementary reporting framework, GROVE (Hillen et al., 2025), future work could strengthen vignette validation with brief age-appropriate realism ratings, pilot testing or cognitive interviewing, expert review, and direct checks of the intended contextual contrast. These additions would complement the present literature-based construction and transparent reporting of the vignette materials.

A further measurement limitation concerns the social-cognitive measures used in Study 2b. The Chinese BES adaptation was originally developed in adolescent samples rather than specifically validated for 8–10-year-old children. Although internal consistency was acceptable in the present sample, age-specific construct validity was not independently established. The ToM measure also comprised only two second-order false-belief items, yielding a restricted 0–2 score. The observed distribution did not show a pronounced ceiling pattern, and categorical sensitivity analyses supported the same conclusions, but the limited score range constrains measurement precision. Future research should use broader age-validated measures of empathy and ToM in middle childhood.

Finally, participants were recruited from schools within a single cultural background in China. The generalizability of the observed responses to cooperation, competition, regulatory urgency, and specific IER strategies therefore remains to be established across cultural settings. Cross-cultural research would help determine which aspects of context-sensitive IER are broadly shared and which depend on culturally shaped interpersonal norms (López-Pérez & Pacella, 2021).

Taken together, the findings make three complementary theoretical contributions. First, they support a capacity-based account of children’s IER: general cognitive-control capacity and social cognition were systematically associated with interpersonal regulatory tendencies. Second, they support a contextual account: children modified both the emotional outcomes they sought and the strategies they preferred as social goals and situational demands changed. Third, the person-by-context findings indicate that these two levels cannot be treated independently, because individual capacities were selectively associated with children’s responsiveness to contextual variation. Viewed together, these contributions support an integrative developmental model of child-to-peer IER in which regulator-level capacities and social-context inputs jointly shape the cycle of identifying emotion, appraising need, selecting a response, and implementing it. Accordingly, the development of IER may be better understood not simply as the acquisition of increasingly adaptive strategies, but as the emerging ability to coordinate cognitive capacity with the goals and demands of an interpersonal situation. In this sense, the central developmental shift is from possessing regulatory capacity to deploying that capacity in a context-sensitive manner.

## 7. Conclusions

The present research provides an integrated account of interpersonal emotion regulation in middle childhood by linking age-related variation, cognitive and social-cognitive capacities, and sensitivity to interpersonal context. Across four studies, children’s IER showed systematic age-related variation and was associated with executive function, empathy, and theory of mind. Children also adjusted their regulatory decisions in response to changes in social goals and regulatory demands. At the person-by-context level, general cognitive-control capacity was associated with responsiveness to regulatory goals, whereas social cognition was associated with demand-related variation in cognitive engagement.

Taken together, these findings suggest that the development of IER involves more than acquiring a repertoire of increasingly adaptive regulatory strategies. It also involves coordinating regulatory capacities with what an interpersonal situation requires. The integrative model proposed here summarizes this developmental pattern by linking general cognitive-control capacity, social cognition, regulatory goals, and regulatory demands within a common interpersonal regulation cycle. By integrating capacity-based, motivational, and context-sensitive perspectives, the present research highlights the importance of considering both the child and the situation when explaining how children regulate others’ emotions. More broadly, understanding IER development may require moving from the question of which strategies children can use toward the question of how developing capacities are deployed in relation to interpersonal goals and situational demands.

## Figures and Tables

**Table 1:** Continuous age associations with adaptive interpersonal emotion regulation.

IER Outcome	N	*M* (*SD*)	*B*/12 Months	95% CI	Std. β	*R* ^2^	*p*	BH-FDR q
Cognitive engagement	220	21.50 (4.85)	2.05	[1.42, 2.69]	0.379	0.144	<0.001	<0.001
Affective engagement	219	23.45 (4.05)	1.79	[1.23, 2.35]	0.396	0.157	<0.001	<0.001
Distraction	220	21.87 (5.48)	0.27	[−0.55, 1.09]	0.044	0.002	0.519	0.519
Overall adaptive IER ^†^	219	66.84 (11.29)	4.09	[2.55, 5.63]	0.324	0.105	<0.001	—

Note: ^†^ Secondary composite outcome; not included in the six-test BH-FDR correction family.

**Table 2:** Continuous age associations with maladaptive interpersonal emotion regulation.

IER Outcome	N	*M* (*SD*)	*B*/12 Months	95% CI	Std. β	*R* ^2^	*p*	BH-FDR q
Suppression	220	11.14 (2.88)	0.27	[−0.14, 0.68]	0.083	0.007	0.199	0.239
Avoidance	220	9.70 (3.34)	−0.84	[−1.33, −0.34]	−0.224	0.050	<0.001	0.002
Co-rumination	220	8.53 (3.84)	−0.81	[−1.37, −0.24]	−0.188	0.035	0.005	0.007
Overall maladaptive IER ^†^	220	29.36 (7.69)	−1.38	[−2.49, −0.26]	−0.160	0.026	0.016	—

Note: ^†^ Secondary composite outcome; not included in the six-test BH-FDR correction family.

**Table 3:** Age-adjusted associations between executive-function components and interpersonal emotion regulation.

Executive-Function Component	B (95% CI)	Std. β	R^2^	ΔR^2^	BH-FDR q
**Panel A. Adaptive IER**					
Inhibitory control	3.58 [0.78, 6.38]	0.260	0.130	0.051	0.015
Working memory	5.03 [2.64, 7.42]	0.390	0.164	0.113	<0.001
Cognitive flexibility	3.35 [2.05, 4.64]	0.433	0.269	0.144	<0.001
**Panel B. Maladaptive IER**					
Inhibitory control	−2.38 [−4.10, −0.65]	−0.275	0.169	0.057	0.010
Working memory	−1.87 [−3.46, −0.28]	−0.231	0.128	0.040	0.021
Cognitive flexibility	−2.33 [−3.49, −1.17]	−0.416	0.247	0.133	<0.001

**Table 4:** Fit and standardized parameter estimates for the latent executive-function models.

Panel A. Model Fit							
**Outcome**	**χ^2^ (df)**	***p***	**CFI**	**TLI**	**RMSEA (90% CI)**	**SRMR**	**R^2^**
Adaptive IER	3.09 (2)	0.213	0.990	0.971	0.066 [0.000, 0.202]	0.024	0.389
Maladaptive IER	3.35 (2)	0.187	0.988	0.964	0.074 [0.000, 0.207]	0.025	0.359
**Panel B. Standardized Measurement and Structural Parameters**							
**Outcome**	**Parameter**		**Std. Estimate**	**SE**	**95% Wald CI**		***p***
Adaptive IER	Inhibitory control loading		0.643	0.072	[0.502, 0.784]		<0.001
	Working memory loading		0.678	0.069	[0.543, 0.813]		<0.001
	Cognitive flexibility loading		0.732	0.066	[0.603, 0.861]		<0.001
	EF → adaptive IER		0.623	0.072	[0.482, 0.764]		<0.001
Maladaptive IER	Inhibitory control loading		0.644	0.072	[0.503, 0.785]		<0.001
	Working memory loading		0.677	0.070	[0.540, 0.814]		<0.001
	Cognitive flexibility loading		0.733	0.067	[0.602, 0.864]		<0.001
	EF → maladaptive IER		−0.600	0.075	[−0.747, −0.453]		<0.001

**Table 5:** Age-adjusted associations between social-cognitive capacities and interpersonal emotion regulation.

Social-Cognitive Predictor	B (95% CI)	Std. β	R^2^	ΔR^2^	BH-FDR q
**Panel A. Adaptive IER**					
Cognitive empathy	0.71 [0.46, 0.95]	0.349	0.217	0.108	<0.001
Affective empathy	0.88 [0.70, 1.06]	0.546	0.382	0.274	<0.001
Theory of mind	7.34 [5.51, 9.16]	0.468	0.220	0.195	<0.001
**Panel B. Maladaptive IER**					
Cognitive empathy	−0.21 [−0.39, −0.03]	−0.169	0.059	0.026	0.019
Affective empathy	−0.21 [−0.34, −0.08]	−0.212	0.074	0.041	0.002
Theory of mind	−2.08 [−3.43, −0.74]	−0.216	0.050	0.041	0.003

**Table 6:** Moderation of regulatory-goal responsiveness by individual capacities.

Individual Capacity	Omnibus Wald χ^2^(2)	*p*/BH-FDR q	Aggression-Oriented Interaction	Approach-Oriented Interaction
General cognitive-control capacity	7.45	0.024/0.048	B = −0.51; OR = 0.60 [0.24, 1.52]; Holm *p* = 0.282	B = −1.48; OR = 0.23 [0.06, 0.83]; Holm *p* = 0.049
Social cognition	0.01	0.997/0.997	B = −0.02; OR = 0.98 [0.48, 1.99]; Holm *p* = 1.000	B = 0.04; OR = 1.04 [0.34, 3.18]; Holm *p* = 1.000

**Table 7:** Capacity × regulatory-demand interactions across IER strategies.

Individual Capacity × Demand	Cognitive Engagement	Affective Engagement	Distraction	Suppression
General cognitive-control capacity × demand	−0.57 [−2.11, 0.97] q = 0.622	−0.73 [−2.34, 0.87] q = 0.622	1.08 [−0.58, 2.75] q = 0.536	−0.37 [−1.25, 0.52] q = 0.622
Social cognition × demand	−1.97 [−3.35, −0.60] q = 0.039	−0.25 [−1.45, 0.96] q = 0.740	0.29 [−1.44, 2.03] q = 0.740	−0.61 [−1.39, 0.16] q = 0.489

## Data Availability

The data presented in this study are available from the corresponding author on reasonable request. The data are not publicly available because the participants were minors and the informed-consent materials did not authorize unrestricted public sharing of individual-level data; access is therefore subject to ethical and privacy safeguards.

## Supplementary Materials

The following supporting information can be downloaded at: https://www.mdpi.com/article/10.3390/bs16101862/s1.

## Abbreviations

The following abbreviations are used in this manuscript:

IER	Interpersonal emotion regulation
ToM	Theory of mind
