# Supplementary material for: Interpersonal Emotion Regulation in Middle Childhood: From Cognitive Capacity to Context-Sensitive Regulation
Source: Behav Sci (Basel). 2026 Oct 9;16(10):1862. doi: 10.3390/bs16101862 (PMC13701323; doi:10.3390/bs16101862)
Supplement: Supplementary file 1 [file behavsci-16-01862-s001.zip › behavsci-4543898-supplementary.pdf]

Supplementary Materials

Interpersonal Emotion Regulation in Middle Childhood: From Cognitive Capacity to Context-Sensitive Regulation

Contents

Supplementary Material S1. Study 1 interpersonal emotion regulation vignette and administration
Supplementary Material S2. Representative regulatory-response items
Supplementary Material S3. Theory-of-mind task: two second-order false-belief stories
Supplementary Material S4. Study 3b regulatory-demand manipulation
References

Supplementary Material S1. Study 1 interpersonal emotion regulation vignette and administration

Task basis. The vignette-based interpersonal emotion regulation (IER) assessment was adapted from Pauw et al. (2019) and Rose and Asher (2004). The vignette used in the study is presented below.

S1.1. Social-stress vignette

> *Imagine that one day your best friend has to give a presentation in front of the whole class. When your friend stands in front of the class, your friend forgets what they wanted to say, so the presentation goes very poorly. During the presentation, you notice several classmates quietly laughing at your friend. You see your friend looking at the classmates who are whispering and laughing at them. When the presentation is over and your friend returns to their seat, those classmates continue laughing and talking, and begin pointing at and making fun of your friend. Later, it is time to leave school. You usually spend time with this best friend after school.*

S1.2. Emotion-identification question

Question: How do you think your best friend is feeling?

Response options: happy, sad, afraid, angry, surprised.

S1.3. Regulatory-response prompt and rating scale

Prompt: Your best friend has experienced this. What would you like to do for your friend after school?

Rating scale: Each regulatory response was rated on a 7-point Likert-type scale from 1 (not at all) to 7 (very much).

Supplementary Material S2. Representative regulatory-response items

Representative items for each of the six strategy categories used in the IER task are shown below. Each item used the response format described in S1.3.

Classification	Strategy	Representative item
Adaptive	Cognitive engagement	To what extent would you give your friend some advice about how to avoid being made fun of by the other classmates?
Adaptive	Affective engagement	To what extent would you comfort your friend and tell them that this kind of thing could happen to anyone?
Adaptive	Distraction	To what extent would you ask your friend whether they would like to play a game with you or do something fun?
Maladaptive	Suppression	To what extent would you encourage your friend to suppress their feelings?
Maladaptive	Avoidance	To what extent would you act as if the other classmates' teasing of your friend had never happened?
Maladaptive	Co-rumination	To what extent would you tell your friend that it was their own fault that the other children made fun of them?

Note. The items are representative examples for each strategy category. All items used the 7-point response scale described in S1.3.

Supplementary Material S3. Theory-of-mind task: two second-order false-belief stories

Task basis. Theory of mind (ToM) was assessed with two second-order false-belief stories based on Astington et al. (2002). Story order was counterbalanced across participants.

S3.1. Story 1: Birthday surprise

Huahua's mother wants to give Huahua a puppy as a birthday present. To make the gift a surprise, the mother hides the puppy in the basement and tells Huahua that she will receive a very nice toy doll for her birthday. When Huahua goes to the basement to get her roller skates, she accidentally sees the puppy.

Control questions:

1. Did Huahua see the puppy? *[Expected answer: Yes]*

2. What does Huahua think she will receive for her birthday? *[Expected answer: A puppy]*

3. Does Huahua's mother know that Huahua saw the puppy in the basement? *[Expected answer: No]*

Target question: What does Huahua's mother think Huahua will tell her friend she received for her birthday? *[Expected answer: a toy doll]*

S3.2. Story 2: The crayons

Zhuangzhuang puts the crayons in the desk and leaves the room. Meimei wants to trick Zhuangzhuang, so she takes the crayons from the desk and puts them in a basket. While Meimei is busy hiding the crayons, Zhuangzhuang happens to watch her through the doorway.

Control questions:

1. Did Zhuangzhuang see Meimei? *[Expected answer: Yes]*

2. Where does Zhuangzhuang think the crayons are? *[Expected answer: In the basket]*

3. Does Meimei think Zhuangzhuang can see her? *[Expected answer: No]*

Target question: Where does Meimei think Zhuangzhuang will look for the crayons when he returns to the room? *[Expected answer: in the desk]*

S3.3. Administration and scoring

After the control questions were answered correctly, the target question was administered. A correct answer to each target question received 1 point and an incorrect answer received 0 points. The total ToM score therefore ranged from 0 to 2, with higher scores indicating better second-order false-belief understanding.

Supplementary Material S4. Study 3b regulatory-demand manipulation

The regulatory-demand manipulation used a modified version of the Study 1 vignette. In the low-demand condition, the stressful classroom event ended at the end of the school day, leaving the target ample time before any subsequent task. In the high-demand condition, the same stressful event was followed by an imminent examination, thereby increasing the urgency of down-regulating the target's negative emotion.

After the vignette, children rated four IER strategies - cognitive engagement, affective engagement, distraction, and suppression - using the same 7-point scale from 1 (not at all) to 7 (very much).

References

Astington, J. W., Pelletier, J., & Homer, B. (2002). Theory of mind and epistemological development: The relation between children's second-order false-belief understanding and their ability to reason about evidence. New Ideas in Psychology, 20(2-3), 131-144. https://doi.org/10.1016/S0732-118X(02)00005-3

Pauw, L. S., Sauter, D. A., van Kleef, G. A., & Fischer, A. H. (2019). Stop crying! The impact of situational demands on interpersonal emotion regulation. Cognition and Emotion, 33(8), 1587-1598. https://doi.org/10.1080/02699931.2019.1585330

Rose, A. J., & Asher, S. R. (2004). Children's strategies and goals in response to help-giving and help-seeking tasks within a friendship. Child Development, 75(3), 749-763. https://doi.org/10.1111/j.1467-8624.2004.00704.x
